# CtBP promoted P4HB mediates SLC7A11 maturation to confer ferroptosis resistance in triple-negative breast cancer

**DOI:** 10.1186/s13046-026-03727-1

**Published:** 2026-05-16

**Authors:** Lifen Wang, Hongxia Xu, Tianyu Liu, Dongliang Zhu, Liping Chen, Tzu-ming Liu, Shuhai Lin, Xueyi Liu, Hao Feng, Li Wang, Li-jun Di

**Affiliations:** 1 Department of Biological Sciences of Faculty of Health Sciences, Ministry of Education Frontiers Science Center for Precision Oncology (FSCPO), University of Macau, Macau, China; 2 Present Address: The Fifth Affiliated Hospital of Zunyi Medical University, Zhuhai, Guang Dong Province China; 3 Present Address: Animal Experiment Center, Guangzhou University of Chinese Medicine, Guangzhou, Guang Dong Province China; 4 State Key Laboratory of Cellular Stress Biology, School of Life Science, Faculty of Medicine and Life Sciences, Xiamen University, Xiamen, Fu Jian Province China

**Keywords:** Ferroptosis, Triple negative breast cancer, CtBP, P4HB, SLC7A11

## Abstract

**Background:**

Triple-negative breast cancer (TNBC) is characterized by aggressive clinical behaviour and frequent resistance to chemotherapy. While ferroptosis—an iron-dependent form of cell death driven by lipid peroxidation—holds promise as a therapeutic strategy, its efficacy is often limited by adaptive resistance mechanisms that remain incompletely understood. C-terminal binding protein (CtBP) is an oncogene associated with poor prognosis in TNBC; however, whether CtBP confers resistance to oxidative stress and ferroptosis, thereby contributing to its oncogenic function, remains unknown.

**Methods:**

We utilized both in vitro cell lines and in vivo mouse models to investigate the role of CtBP in regulating oxidative stress and ferroptosis sensitivity. Integrative analyses, including transcriptomics, metabolomics, and clinical data interrogation, were employed to elucidate the mechanisms underlying CtBP-mediated resistance. Key mechanistic findings were further validated in clinical TNBC specimens using protein correlation analysis.

**Results:**

We identified CtBP as a stress-inducible gene that is essential for ferroptosis resistance in TNBC. Mechanistically, CtBP promotes resistance by transcriptionally upregulating Prolyl 4-Hydroxylase Subunit Beta (P4HB). P4HB, localized to the endoplasmic reticulum (ER), functions as a chaperone to facilitate the correct folding and membrane maturation of SLC7A11, the catalytic subunit of the system xc^-^  cystine/glutamate antiporter pivotal for glutathione (GSH) biosynthesis. We demonstrated that P4HB-mediated SLC7A11 maturation relies on its disulfide isomerase activity and ER retention signal (KDEL). Depletion of either CtBP or P4HB impaired SLC7A11 membrane trafficking, lead to accumulation of lipid peroxides and sensitization to ferroptosis. Conversely, P4HB overexpression restored SLC7A11 function and ferroptosis resistance in CtBP-deficient cells. Furthermore, significant positive correlations among CtBP, P4HB, and SLC7A11 expressions in clinical samples underscore the critical role of CtBP/P4HB/SLC7A11 axis in driving cancer aggressiveness. In vivo, targeting the CtBP/P4HB/SLC7A11 axis sensitized TNBC xenografts to the ferroptosis inducer RSL3, significantly suppressing tumor growth and lung metastasis.

**Conclusions:**

This study identifies the CtBP/P4HB/SLC7A11 axis as a critical mediator of ferroptosis resistance in TNBC. These findings highlight this pathway as a potent therapeutic target to overcome resistance and enhance the efficacy of ferroptosis-inducing regimens in TNBC treatment.

**Supplementary Information:**

The online version contains supplementary material available at 10.1186/s13046-026-03727-1.

## Background

A distinguishing feature of cancer cells is the accumulation of reactive oxygen species (ROS), which contributes to tumor progression, metastasis, and therapy resistance [1]. Elevated ROS levels are accompanied by enhanced antioxidant capacity, supported by metabolic reprogramming that increases the intracellular supply of reducing equivalents such as NADPH and FADH₂, along with the increased synthesis of glutathione (GSH), a major cellular antioxidant [2]. Targeting the redox balance of cancer cells has emerged as a promising therapeutic strategy. Approaches include the use of pro-oxidant agents such as doxorubicin, ROS-generating nanomaterials, and physical approaches like infrared or ultrasound therapies, as well as inhibitors of antioxidant pathways.

### Ferroptosis resistance in cancer

The induction of ferroptosis has emerged as an attractive strategy for cancer treatment. As a distinct form of cell death from apoptosis or necrosis, ferroptosis is characterized by lipid peroxidation, free iron ion accumulation, increased intracellular oxidation, and impaired antioxidant pathways. Ferroptosis in cancer cells is critically dependent on the iron-catalyzed Fenton reaction, where ferrous iron (Fe^2^⁺) promotes the generation of ROS. This surge in intracellular ROS triggers extensive lipid peroxidation—affecting both the plasma membrane and intracellular organelle membranes—that ultimately leads to cell death. By exploring the iron-dependent oxidative vulnerability of cancer cells, ferroptosis offers a promising strategy for selective tumor eradication [3].

However, how to overcome ferroptosis resistance is a challenge in cancer therapy. Although the essence of ferroptosis is the peroxidation of membrane lipids exceeding the tolerable threshold, leading to membrane rupture, but the regulation of ferroptosis lacks a singular core mechanism, displaying significant variability influenced by diverse factors across cell types [4]. For instance, lipid peroxidation is driven by various sources like hydroxyl radicals (HO·), hydroperoxyl radicals (HOO·) from redox enzymes, and peroxides from the mitochondrial electron transport chain (ETC) etc. Each of these factors can induce lipid damage independently, suggesting the lack of a common mechanism in ferroptosis induction. Therefore, the formation of ferroptosis resistance could involve different mechanisms too.

Several mechanisms have been well documented to counteract ferroptosis in mammalian cells. The first one is known as GPX4-GSH System. Within this system, GPX4, a selenoprotein, reduces lipid hydroperoxides to alcohols by oxidizing glutathione (GSH), thereby protecting cells from damage [5]. GSH, a tripeptide composed of glycine, glutamate, and cysteine, is crucial for this process, with cysteine being a key limiting factor for synthesis of GSH. Cysteine is the reduced form of cystine and cystine level is primarily regulated by a transporter complex on the cell membrane consisting of SLC7A11 and SLC3A2 (also known as xCT). Therefore, the SLC7A11-GSH-GPX4 axis is believed to be the primary intracellular defense mechanism against ferroptosis. The other ferroptosis resistance system is known as FSP1-CoQH2 System [6]. FSP1 is a cell membrane-localized NAD(P)H-dependent oxidoreductase, reduces coenzyme Q (CoQ) to CoQH2. CoQH2 can trap lipid peroxyl radicals to inhibit ferroptosis. Since CoQH2 has been discovered outside of mitochondria, this FSP1-CoQH2 system is believed to prevent ferroptosis in cytoplasm. In addition to these well-established mechanisms, recent reports have highlighted novel pathways contributing to ferroptosis resistance. For example, DHODH, situated in the inner mitochondrial membrane, mitigates lipid peroxyl radical accumulation within mitochondria [7]. Another example is the MBOAT1/MBOAT2 system which remodels cell membrane PUFA and MUFA content independent of GPX4 and FSP1 [8]. Gut bacteria also increase ferroptosis resistance in digestive tumors [9]. Oncogenes like HIF1α is also known to promotes ferroptosis resistance [10]. Thus, understanding these compensatory regulators and their tumor-specific associations are important for developing ferroptosis induction as an efficient way to eradicate cancer cells.

### CtBP promotes cancer progression

The C-terminal binding proteins (CtBP1/2) are a pair of proteins that share 70% of homology. Earlier studies mainly discovered the important roles of CtBP in development by applying different animal models [11, 12]. Detailed characterization of CtBP identified several functional domains including the PxDLS binding domain and dehydrogenase domain. Via its PxDLS binding cleft or RRT binding cleft, CtBP forms complexes with multiple epigenetic modifiers such as HMTs (G9a), HDACs (HDAC1 and HDAC2), LSD1, CoREST etc. and plays critical roles in regulating gene expressions [13]. In cancer development, CtBP was reported to regulate the cancer initiation [14–16], cancer metastasis [17–19], the intracellular metabolism [17, 20–24] and drug resistance [25, 26]. CtBPs were shown to transcriptionally repress the expression of multiple tumor suppressor genes, including p16, p21, E-cadherin, SIRT4, SREBF2, RAD51, BRCA1 and PTEN etc. The pan-cancer study also supports CtBP as a cancer risk gene and high expression of CtBP is associated with a worse outcome of patients. The high expression of CtBP in tumors has been observed in various types of cancers [14–18, 20–24]. Therefore, some studies have attempted to develop drugs targeting CtBP for cancer therapy [27, 28, 29, 30].

While the dehydrogenase domain remains elusive about its enzymatic substrate, this domain shows its critical role in making CtBP an intracellular metabolic status sensor by providing NADH or NAD docking site. Several studies have demonstrated the dynamics of CtBP dimerization status influenced by binding to NADH or NAD with NADH showing 100 times more activity to mediate the CtBP dimerization [20, 31–34]. Importantly, Changes in the dimerization status of CtBP in response to metabolic status ultimately manifest as alterations in its transcriptional regulatory activity. Intriguingly, its downstream target genes include numerous regulators of metabolic pathways—such as *SIRT4* and *SREBF2* [17, 24, 35]. Notably, the ability of CtBP to regulate metabolic genes is tightly linked to its role in promoting cancer cell progression and metastasis.

In this study, we found that CtBP is upregulated in response to oxidative stress and promotes resistance to ferroptosis induction. We identified that P4HB is upregulated by CtBP and mediates the ferroptosis-resistant function of CtBP by facilitating the correct folding and membrane localization of SLC7A11. Collectively, our findings reveal that the CtBP-P4HB-SLC7A11 axis is a key mechanism underlying resistance to ferroptosis induction, and we confirmed via in vivo experiments that targeting this pathway effectively enhances the sensitivity of cancer cells to ferroptosis inducers.

## Materials and methods

**Table:** 

REAGENT or RESOURCE	SOURCE	IDENTIFIER
Antibodies		
Anti-GAPDH	Proteintech	10,494—1-AP
Anti-CtBP2	BD Bioscience	612,044
Anti-CtBP1	BD Bioscience	612,042
Anti-P4HB	proteintech-	11,245—1-AP
Anti-SLC7A11	Proteintech	26,864—1-AP
Anti-SOD1	Proteintech	67,480—1-Ig
Anti-SOD2	Proteintech	66,474—1-Ig
Anti-GCLC	Proteintech	12,601—1-AP
Anti-GPX4	Abcam	ab125066
Anti-ACSL4	Proteintech	22,401—1-AP
Anti-FSP1	Proteintech	20,886—1-AP
Anti-DHODH	Proteintech	14,877—1-AP
Anti-4-HNE	Abcam	ab46545
Anti-Na +/K + ATPase	CST	3010S
Anti-ERO1-Lα	CST	3264
Anti-HA-Tag	Proteintech	66,006—2-Ig
Anti-MYC-Tag	Proteintech	16,286—1-AP
Chemicals		
N-Acetylcysteine	Beyotime	ST-1546
ZVAD-FMK	MCE	HY-16658B
Necrostatin-1	MCE	HY-15760
Ferrostatin-1	MCE	HY-100579
Erastin	MCE	HY-15763
RSL3	Selleck	S8155
Cycloheximide	Selleck	S7418
MG132	MCE	HY-13259
TCEP hydrochloride	MCE	W011500
Bacitracin	MCE	HY-107193
Hydrogen peroxide 30%	Merck Millipore	107,209
TBHP	Aladdin	B106035

### Cell culture and transfection

Human breast cancer cell line MDA-MB-468, MDA-MB-231, MDA-MB-436, HCC1954, BT549, T47D and MCF-7, human cervical cancer line HeLa, human liver cancer cell line Huh7, human embryonic kidney cell line HEK293T were cultured at 37℃ and 5% CO2 in Dulbecco’s modified Eagle medium (DMEM, Gibco) with 2 mM glutamine, 4.5 g/L D-Glucose, 1 mM sodium Pyruvate and 10% fetal bovine serum FBS (Gibco). Plasmid and siRNA (purchased from GenePharma) were transfected using Lipofectamin 3000 Transfection Reagent (Invitrogen, L3000001) according to the product manual.

### Expression and knockdown vectors

P4HB, P4HB-MUT, P4HB-ΔKDEL, CtBP2 coding sequence were cloned into pCDH-CMV (Addgene, #72,265) expression vector. The shRNAs targeting ERO1A, CtBP1/2 were cloned into pLKO.1(Addgene, #8453). The lentiCRISPR v2 -puro (Addgene, #52,961) were used for cloning CtBP1/2 and P4HB. Primers are listed in table S1.

### Lentivirus package and stable cell line construction

Plasmids were co-transfected with pDelt and pVSVG in a ratio of 4:2:3 into 293 T cells. The supernatant containing virus was collected after 48 h, after centrifuged in 500 g for 10 min, the supernatant was incubated with virus concentration using a commercial concentrator (Takara, #631,231) for overnight. After centrifugation at 1500 g for 45 min, disturbed supernatant and gently resuspend the pellet in one milliliter using complete DMEM. Subsequently, cells were transfected with the virus and cultured in the presence of 8 μg/ml polybrene (Beyotime, C0351) for 24 h. Thereafter, cells were selected with the optimal concentration of puromycin (Beyotime, ST551) to obtain stable cell lines. The efficiency of overexpression or knockout was confirmed by RT-qPCR and Western blot analysis.

### RNA isolation and Real-time qPCR

RNA was extracted using TRIzol reagent (Invitrogen, 15,596,018), following determined mRNA concentration using a NanoDrop. 500 ng of mRNA was reversed to cDNA using reverse transcriptase (Takara, RR036B) according to manufacturer’s instructions. The cDNA was tenfold diluted before performed RT-qPCR analysis. RT-qPCR was performed using Power SYBR Green PCR Master Mix (Transgen biotech, S10521), PCR amplified using the Bio-Rad qPCR system. CT values of each gene were normalized to the internal control gene β-Actin. The relative expression of the indicated genes was calculated using the ΔΔCt method. Primers are listed in table S1.

### Immunoprecipitation (IP) and Western blot

Cells were scraped off from 10 cm dishes, the whole cell lysates were extracted by IP buffer (Thermo fisher, 87,788) with protease inhibitor cocktail (CST, #5871). The protein samples were collected from the supernatant after centrifugation at 12,000 g for 15 min at 4℃ and then quantified using the BCA Protein Assay Kit (Thermo fisher, pierce 23,227). The protein solutions were incubated with the indicated antibodies in protein A/G beads (Millipore, IP10) and rotated at 4℃ overnight. After washing 3 times, the precipitates were resuspended with SDS-PAGE loading buffer (Bio-Rad, 1,610,747) and boiled for 10 min. After centrifugation at 12,000 rpm for 5 min, the supernatant was collected for SDS-PAGE analysis.

Cells were collected and lysed by RIPA buffer (Thermo fisher, 89,901) containing protease inhibitor and quantified by bicinchoninic acid assay. 20 µg of protein was separated on a 12% bis–tris gradient gel following transferred to nitrocellulose (NC) membranes for 1 h at 380 mV in ice-cold tris–glycine transfer buffer containing 20% methanol. After being blocked with 5% non-fat dry milk, the NC membranes were incubated with the indicated primary antibodies overnight. The HRP-conjugated, species-specific secondary antibodies (Sentacruz, sc—516,102) were used to incubate the membranes on the following morning for 1 h at room temperature. The target bands were detected using enhanced chemiluminescence on a ChemiDo and the signal was analyzed using Image J software.

### Membrane and cytosol protein extraction

Cells were scraped off from 15 cm dishes and collected by centrifugation. The pellet was resuspended in ice-cold PBS, with a portion taken for counting. The remaining cells were centrifuged at 600 g for 5 min at 4℃, then again for 1 min to remove residual liquid. One milliliter of membrane protein extraction reagent A (Beyotime, #P0033), with added PMSF, was added to the cells, which were then placed in an ice bath for 10—15 min. The samples were freeze-thawed three times between liquid nitrogen and room temperature, and a small portion was checked under a microscope for cell breakage. After centrifugation at 700 g for 10 min at 4℃, the supernatant was carefully collected. The samples were then centrifuged at 14,000 g for 30 min at 4℃ to precipitate cell membrane debris. The supernatant, containing cytoplasmic protein, was collected. The pellet was resuspended in 200 µl of membrane protein extraction reagent B, vortexed, and placed in an ice bath. This process was repeated to fully extract membrane proteins. Finally, the samples were centrifuged at 14,000 g for 5 min at 4℃, and the supernatant was collected as the membrane protein solution.

### ChIP

Chromatin immunoprecipitation (ChIP) was performed as follows: Cells were cross-linked with 1% formaldehyde at 37 °C for 10 min and quenched with 0.125 M glycine at room temperature for 5 min. After PBS washing, cells were collected by centrifugation (2,000 rpm, 4 °C) and lysed in low-salt IP buffer. Chromatin was sheared by sonication (30 s on/30 s off, 12—15 cycles, 30—40% amplitude, 4 °C). The clarified lysate was centrifuged (15,000 rpm, 4 °C, 20 min), and 20 μl was retained as input. The remaining lysate was incubated with 5 μg of indicated antibody overnight at 4 °C with rotation, followed by addition of 25 μl Protein A/G agarose beads for 2 h. Immunocomplexes were sequentially washed with high-salt buffer (4 ×), low-salt buffer (1 ×), and TE buffer (2 ×). Beads were resuspended in elution buffer containing proteinase K and reverse cross-linked at 65 °C overnight. DNA was extracted by phenol–chloroform, ethanol precipitated, and dissolved in 50 μl water for RT-PCR analysis. Chip-qRCR Primer Sequence: 1F:tacgcggtccagtcagaatg; 1R:ctcattggctcccgacaaga; 2F:catcccttggccaatcagga; 2R:cgctcgctgtctttcttagc; 3F:ggaaattgcgtcaccccaac; 3R:tttcggtagcacggactctg.

### Dual-luciferase reporter assay

293FT cells were plated in 24-well plates (2 × 10^5^ cells/well) and incubated for 24 h to achieve 70–80% confluency. Cells were co-transfected with 400 ng of either pGL3-basic vector or P4HB promoter-driven firefly luciferase reporter construct, together with 40 ng of Renilla luciferase control vector (pRL-TK), using Lipofectamine 3000 (Invitrogen, L3000001) according to the manufacturer's protocol; experimental groups were simultaneously subjected to CtBP overexpression or knockdown. At 48 h post-transfection, cells were rinsed with PBS and lysed in 100 μl of 1 × Passive Lysis Buffer (Promega, E1910) with gentle agitation for 15 min at ambient temperature. 20 μl of clarified lysate were subjected to sequential addition of Luciferase Assay Reagent II (LAR II) and Stop & Glo® Reagent for quantification of firefly and Renilla luciferase activities, respectively, using a microplate luminometer. Relative promoter activity was expressed as the normalized firefly/Renilla (F/R) luminescence ratio.

### Immunofluorescence staining and microscope imaging

Cells were seeded at 50% confluence into confocal dishes; cells were pretreated with specific drug for 24 h the next day. After fixed in 4% paraformaldehyde (PFA) for 15 min, 0.5%Triton X-100 were used to permeabilize for 20 min before washing three times with PBS. Next, the cells were blocked with 1% bovine serum albumin (Sigma, A7030) for 0.5 h at room temperature. Subsequently, cells were incubated with indicated primary antibodies diluted at 4℃ overnight before washing three times with PBS and then incubated with specific secondary antibodies diluted at room temperature for 1 h, followed by three washes with PBS. Finally, cells were mounted with anti-fade mounting media containing DAPI (Beyotime, Cat# P0131) covered by coverslips. Fluorescent images were acquired with LSM710 Confocal microscope (Carl Zeiss) and analyzed in ZEN 2.5 lite software.

### Cell viability assay and cell death measurement

Cells were seeded in 96-well plates in the presence of specific treatment. To assess cell viability, medium containing CCK8 (Beyotime, C0041) was added to each well, following incubated for 1 h. The absorbance was measured at 450 nm, then cell viability was normalized to each group that without treatment. For cell death measurement, we used PI (Beyotime, ST511) staining to detect the dead cell ratio of breast cancer cell lines underwent specific treatment to induce cell death, then detected by flow cytometry and microscope according to the instructions.

### Flow cytometry for ROS

1 × 10^6^ cells were seeded in 12-well dishes, incubated with or without drug treatment for 24 h in respective growth medium. Then the cells were washed with 1 × PBS and stained with 10 uM DCFH-DA (Beyotime, S0035S) for 20 min at 37 °C in free medium. Cells were then washed, trypsinized and resuspended in PBS. Following filtered, analyzed and collected with CytoFLEX Flow Cytometer. Fluorescence was excitated with the 488 nm laser (DCFH-DA) and monitored in the blue 525/40 detector respectively. Data was analyzed by FlowJo v10.

### Determination of lipid peroxidation

1 × 10^6^ cells were seeded in 12-well dishes, incubated with or without drug treatment for 24 h in respective growth medium. Then the cells were washed with 1 × PBS and stained with 5 uM BODIPY 581/591 C11 (Invitrogen, #D3861) for 30 min at 37 °C in HBSS. Cells were then washed, trypsinized and resuspended in PBS. Following filtered, analyzed and collected with CytoFLEX Flow Cytometer. Fluorescence was excitated with the 488 nm laser (BODIPY 581/591 C11) and monitored in the blue 525/40 detector respectively. Data was analyzed by FlowJo v10. At least 10,000 single cells were analyzed per well, and all experiments were performed in triplicate. For the image of lipid peroxidation, cells were staining by BODIPY 581/591 C11 for 30 min at 37 °C in HBSS; and fixed with 4% paraformaldehyde (PFA) for 15 min before washing three times with PBS. Finally, cells were treated with anti-fade mounting media (Beyotime, Cat# P0126) covered by coverslips. After that, images were acquired with LSM710 Confocal microscope (Carl Zeiss). Ratio of lipid peroxidation to non-oxidation is quantified by Image J.

### Detection of glutathione

Cells were collected from 6-well dishes. Cellular total glutathione was detected using total glutathione assay kit (Beyotime, S0052) according to the manufacturer’s instructions. A part of the cells was separated and quantified by bicinchoninic acid assay. Total glutathione was calculated according to the standard curve and normalized to protein concentration.

### Detection of glutathione peroxidase activity

Cells were collected from 6-well dishes. Cellular glutathione peroxidase activity was detected using Cellular Glutathione Peroxidase Assay Kit with NADPH (Beyotime, S0056) according to the manufacturer’s instructions. Cellular glutathione peroxidase activity was calculated according to the standard curve and normalized to protein concentration.

### Cystine uptake assay

MDA-MB-468 cells (1.5 × 10^4^ cells/well) were plated in 96-well plates and incubated overnight at 37 °C with 5% CO₂. Following serum starvation and cystine deprivation, cells were washed thrice with pre-warmed cystine-free, serum-free DMEM (Gibco, 21,013,024) and equilibrated for 5 min. Cystine uptake was subsequently initiated by addition of CA Uptake Solution (Dojindo, UP05) or vehicle control (Blank) for 30 min. Cells were then rinsed thrice with ice-cold PBS, lysed in methanol, and reacted with Working Solution at 37 °C for 30 min. Fluorescence intensity was quantified by microplate fluorimetry (Ex/Em = 490/535 nm).

### RNA sequencing analysis

Total RNA was extracted from MDA-MB-468 cells using RNeasy Plus Mini Kit (Qiagen, 74,034) after treatment. A data quality check was done on Agilent 5400. cDNA libraries are sequenced by Novogene (Beijing, China) using Illumina NovaSeq platforms. The sequence reads were mapped to the latest UCSC transcript set, and the gene expression level was estimated by FeatureCounts. VST (the variance stabilizing transformation) was used to normalize gene expression. DESeq2 program was used to identify differential expressed genes (DEG) through Gene Ontology (GO) pathway and KEGG pathway (Kyoto Encyclopedia of Genes and Genomes) using ClusterProfiler package. Genes showing altered expression with p adj. < 0.05 and absolute Log2(fold change) > 0.5 were considered as differential expressed genes in CtBP/P4HB knock-down group comparing with control group in MDA-MB-468 cell lines.

### Metabolomics analysis

Untargeted metabolomic analysis was conducted by Calibra Lab at DIAN Diagnostics (Hangzhou, Zhejiang, China) on their CalOmics metabolomics platform. 1 × 10^7^ cells were collected for each sample, followed by washed three times with PBS. A part of cells was used for cell counting and protein concentration detection using BCA assay. Each group was performed in sextuplicate. Samples were extracted using methanol in a ratio of 1:4. The mixtures were shaken for 3 min and precipitated by centrifugation at 4000 × g, 10 min at 20 ℃. Four aliquots of 100 μL supernatant were transferred to sample plates and dried under blowing nitrogen, then re-dissolved in reconstitution solutions for sample injection into UPLC-MS/MS systems. The instruments for the four UPLC-MS/MS methods are ACQUITY 2D UPLC (Waters, Milford, MA, USA) plus Q Exactive (QE) hybrid Quadrupole-Orbitrap mass spectrometer (Thermo Fisher Scientific, San Jose, USA). QE mass spectrometer was operated at a mass resolution of 35,000, the scan range was 70—1000 m/z. In the first UPLC-MS/MS method, QE was operated in positive ESI mode and the UPLC column was C18 reverse-phase (UPLC BEH C18, 2.1 × 100 mm, 1.7 um; Waters); the mobile solutions used in the gradient elution were water (A) and methanol (B) containing 0.05% PFPA and 0.1% FA. In the second UPLC-MS/MS method, QE was operated in negative ESI mode, and the UPLC column was C18 reverse-phase (UPLC BEH C18, 2.1 × 100 mm, 1.7 um; Waters), the mobile solutions used in the gradient elution were water (A) and methanol (B) containing 6.5 mM ammonium bicarbonate at pH 8. The third UPLC-MS/MS method had the QE operated in ESI positive mode and the UPLC column was C18 reverse-phase (UPLC BEH C18, 2.1 × 100 mm, 1.7 um; Waters), the mobile solutions were water (A) and methanol/acetonitrile/water (B) contain 0.05% PFPA and 0.01% FA. In the fourth method, QE was operated in negative ESI mode, the UPLC column was HILIC (UPLC BEH Amide, 2.1 × 150 mm, 1.7 um; Waters), and the mobile solutions were water (A) and acetonitrile (B) with 10 mM ammonium formate.

### Transmission Electron Microscope (TEM) analysis

The samples were fixed in 2.5% glutaraldehyde (pH 7.4) for 2 h. After washed three times with 0.1 M phosphate buffer (pH 7.2) and fixed in 1% osmic acid at 4℃for 2 h. Then the samples were gradient dehydrated in a graded series of ethanol. Subsequently, the samples were embedded in Epon-Araldite resin for penetration and placed in a mold for polymerization. After the semi thin section was used for positioning, the ultrathin section was made and collected for microstructure analysis. Followed the counterstaining of 3% uranyl acetate and 2.7% lead citrate. Then observed with a JEM1400 transmission electron microscope. TEM analysis was conducted by Wuhan MISP Bio-technology CO.

### Animal experiment

Our animal research protocol UMARE-012—2020 has been approved by the UM Animal Research Ethics Committee for the animal experiment.

For the subcutaneous tumor formation experiment, 5 × 10^6^ of MDA-MB-468 was re-suspended with 0.2 ml PBS and inoculated in the middle and back of the axilla of 6–8-week-old NSG mice. Tumor size was measured within 1—2 months, and after carbon dioxide euthanasia, the subcutaneous tumors were removed. The tumors were weighed and photographed before being fixed for immunohistochemical experiments.

For the lung metastasis experiment, 2 × 10^6^ of MDA-MB-468 with Luciferase-GFP stabled expression were suspended with 0.2mLPBS and administered to 6—8 weeks old NSG mice through tail vein slowly and evenly. After 2 month of feeding, the fluorescence signals from metastatic tumors in the lungs of mice were detected by the Animal In Vivo Imaging System (BLT_AniView Phoenix Full Spectrum) five minutes after intraperitoneal injection of the D-Luciferin substrate (YEASEN, #40903ES03). After carbon dioxide euthanasia, the lung tissue was removed and photographed the fluorescence signals to measure tumor metastasis number then fixed for immunohistochemical experiments.

### Hematoxylin and Eosin stain

Immerse slides in xylene for 5 min, repeat twice. Pass through graded alcohols (100%, 95%, 70%) for 5 min each, then rinse in distilled water for 5 min. Place slides in hematoxylin for 5—10 min, rinse in running tap water for 5 min, differentiate in 1% acid alcohol for 10—30 s, rinse again in running tap water for 5 min, and blue in Scott's tap water substitute or distilled water for 1—2 min. Place slides in eosin for 2—3 min, rinse in distilled water for 1 min. Dehydrate through graded alcohols (70%, 95%, 100%) for 2 min each, clear in xylene for 5 min, repeat twice. Mount coverslips using a suitable mounting medium, allow slides to dry, and examine under a microscope.

### Immunohistochemistry

Deparaffinize mouse tissue sections and patient tissue microarray sections (SHANGHAI OUTDO BIOTECH CO., HBre-Duc052Bch-1) by immersing slides in xylene for 5 min, repeating twice. Rehydrate by passing through graded alcohols (100%, 95%, 70%) for 5 min each, followed by distilled water for 5 min. For antigen retrieval, place slides in a pressure cooker with Citrate Antigen Retrieval Solution (Beyotime, #P0081) and heat at 121 °C for 10 min. Allow to cool at room temperature for 20 min, then rinse in PBS. Incubate slides in 3% hydrogen peroxide in PBS for 10 min to quench endogenous peroxidase activity, then block non-specific binding sites with normal blocking BSA for 30 min at room temperature. Dilute the primary antibody (typically 1:100 to 1:1000) and apply to the tissue sections. Incubate in a humidified chamber at 4 °C overnight. Rinse slides in PBS three times for 5 min each. Dilute the secondary antibody (typically 1:200 to 1:1000) and apply to the tissue sections. Incubate for 30 min at room temperature, then rinse in PBS three times for 5 min each. Apply DAB to the tissue sections and incubate for 5—10 min at room temperature. Rinse slides in distilled water. Counterstain with hematoxylin for 1—2 min, then rinse in running tap water for 5 min. Dehydrate through graded alcohols (70%, 95%, 100%) for 2 min each, and clear in xylene for 5 min, repeating twice. Mount coverslips using a suitable mounting medium, allow slides to dry, and examine under a microscope. Pathologists scored different sections on the basis of IHC staining status, with scoring criteria of 0 (unstained), 1 (weakly stained), 2 (moderately stained) and 3 (strongly stained). The H-score value was calculated according to the following formula: H-score = (3 × the percentage of cells scoring 3) + (2 × the percentage of cells scoring 2) + (1 × the percentage of cells scoring 1) + (0 × the percentage of cells scoring 0).

### Ferroptosis score analysis

A total of 242 ferroptosis suppressor genes and 269 driver genes were referenced from FerrDb (http://www.zhounan.org/ferrdb/current/). The gene expression profiles of breast cancer or TNBC patients from the TCGA database were utilized to conduct ferroptosis suppressor and driver score analyses using single-sample Gene Set Enrichment Analysis (ssGSEA). The relative abundances of ferroptosis-related suppressor and driver genes in breast cancer were analyzed in relation to P4HB and CtBP1 through Pearson correlation tests.

### Statistical analysis

Please refer to the legend of figures for sample sizes and statistical tests performed. Data was plotted and analyzed with GraphPad Prism 10 software. Differences were considered statistically significant when the *p*-value was less than 0.05 and otherwise not significant (ns). *P < 0.05, **P < 0.01, ***P < 0.001, and ****P < 0.0001.

## Results

### CtBPs function as oxidative stress inducible factors in breast cancer (BC) cells

**Fig. 1:**
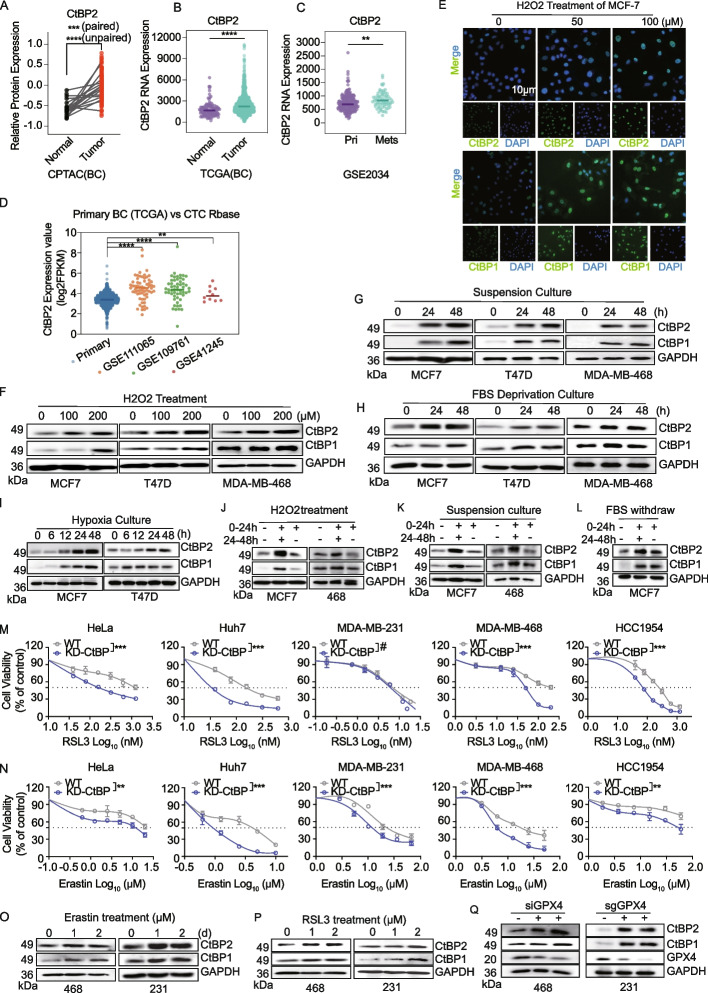
**A** Comparison of CtBP protein levels in breast cancer samples (*n* = 124) and matched normal mammary gland tissues (*n* = 18), analyzed using data from the CPTAC (Breast Cancer) dataset. **B** Comparison of CtBP2 mRNA expression in cancer tissues (*n* = 1096) versus adjacent normal tissues (*n* = 113), based on data from TCGA (Breast Cancer). **C** Analysis of CtBP2 mRNA expression in metastatic tumors (*n* = 69) compared with matched primary tumors (*n* = 217), using data from GSE2034. **D** Comparison of CtBP2 mRNA FPKM levels between primary tumors (retrieved from TCGA-BC, *n* = 1218) and circulating tumor cells (retrieved from ctcRbase, GSE111065 *n* = 63, GSE109761 *n* = 54, GSE41245 *n* = 10). **E** IF and (**F**) WB quantification of CtBP following treatment with the indicated concentrations of H_2_O_2_ for 24 h (*n* = 3). **G**–**I** WB quantification of CtBP under suspension culture (**G**), FBS deprivation (**H**), and hypoxic culture conditions (**I**) (*n* = 3). (**J**–**L**) WB quantification of CtBP in breast cancer cells treated with 200 μM H_2_O_2_ (**J**), suspension culture (**K**), or FBS deprivation (**L**) for 24 h, followed by withdrawal of the treatment and subsequent culturing for another 24 h (*n* = 3). (M, N) Cell viability of HeLa, Huh7, MDA-MB-231, MDA-MB-468, and HCC1954 cells with CtBP KD and treated with Erastin or RSL3 (*n* = 3). (O, P) WB quantification of CtBP in MDA-MB-468 and MDA-MB-231 cells treated with Erastin (10 μM) and RSL3 (5 nM and 100 nM) for 0, 24, and 48 h (*n* = 3). (Q) WB quantification of CtBP and GPX4 upon GPX4 KD (*n* = 3). For (A) P values were calculated using both paired and unpaired two-tailed Student’s t-test. For (**B**), (**C**), (**D**), (**M**), (**N**) P values were calculated using a two-tailed Student’s t-test. #, *P* > 0.05; *, *P* < 0.05, **, *P* < 0.01, ***, *P* < 0.001 and ****, *P* < 0.0001. For (**B**), (**C**), (**D**) data are presented as median with interquartile range; for (M), (N) data are presented as mean ± SD, (n ≥ 3)

Higher grade tumors exhibit an elevated level of oxidative stress, to which cancer cells adapt by upregulating genes involved in stress resistance. CtBP has been implicated in BC progression. Analysis of the CPTAC dataset revealed significantly increased CtBP expression in tumor tissues compared to matched normal mammary gland (MG) tissues (Fig. 1A). Higher expressions of CtBP were also observed in cancer tissues (TCGA dataset, comparing to matched normal MG tissues) and metastatic tumors (GSE2034 dataset, comparing to primary tumors) (Fig. 1B and C). Given the heightened oxidative stress experienced by circulating tumor cells, we examined CtBP expression in single circulating cells from multiple BC patients and found consistent upregulation relative to primary tumors (Fig. 1D).

To determine whether CtBP is responsive to oxidative stress, we exposed cells to stress conditions mimicking the tumor microenvironment, including H₂O₂ treatment (Fig. 1E–F and sFig. 1 A), suspension culture (Fig. 1G and sFig. 1B), serum deprivation (Fig. 1H and sFig. 1 C), and hypoxia (Fig. 1I and sFig. 1D). These conditions induced CtBP expression, even in non-tumorigenic MCF10a cells (sFig. 1E and sFig. 1 F). CtBP levels returned to baseline within 24 h after stress removal (Fig. 1J–L and sFig. 1G). CtBP knockdown (KD) impaired cell growth and increased reactive oxygen species (ROS) accumulation (sFig. 1 J–M), which was restored by the apoptosis inhibitor ZVAD or the antioxidant NAC, but only marginally by the necrosis inhibitor NEC (sFig. 1 N). CtBP KD also sensitized cells to death under oxidative stress conditions (H₂O₂, TBHP, suspension; sFig. 1O–R), with ZVAD and NAC restoring viability under suspension culture (sFig. 1S). Together, these data suggest CtBP is inducible by oxidative stress and contributes to the oxidative stress resistance in BC.

Since ferroptosis induction relies on oxidative stress accumulation, we wonder if CtBP is also involved in ferroptosis resistance. CtBP KD increased sensitivity to ferroptosis inducers RSL3 (GPX4 inhibitor) and Erastin (Xc⁻ transporter inhibitor) across multiple BC cell lines (Fig. 1M–N, sFig. 1 T). Consistently, both Erastin and RSL3 treatment of BC cells could induce the expression of CtBP (Fig. 1O and P). Moreover, GPX4 KD, which resembles RSL3 treatment, also resulted in the upregulation of CtBP (Fig. 1Q). These data suggest CtBP promotes ferroptosis resistance upon its induction by ferroptosis inducers in BC.

### Loss of CtBP resulted in increased oxidative stress and increased ferroptosis

To further investigate the mechanism of CtBP upregulation under stress conditions, we explored changes in both CtBP transcriptional levels and protein stability. We found that the stresses mildly but significantly upregulated the CtBP1 and CtBP2 mRNA (sFig. 2A-sFig. 2 C). Consistently, H_2_O_2_ treatment slightly upregulated CtBP protein levels with presence of MG132, suggesting stresses induce de novo synthesis of CtBP (sFig. 2D). CtBP2 mRNA returned to baseline following reoxygenation after 24 h hypoxia (1% O₂), confirming transcriptional reversibility (sFig. 2E). Notably, CtBP protein levels also increased under H₂O₂ exposure despite cycloheximide (CHX) treatment, indicating reduced degradation contributes to CtBP accumulation (sFig. 2 F). These findings suggest CtBP levels are regulated through both synthesis and degradation, enabling dynamic adaptation to oxidative stress.

**Fig. 2:**
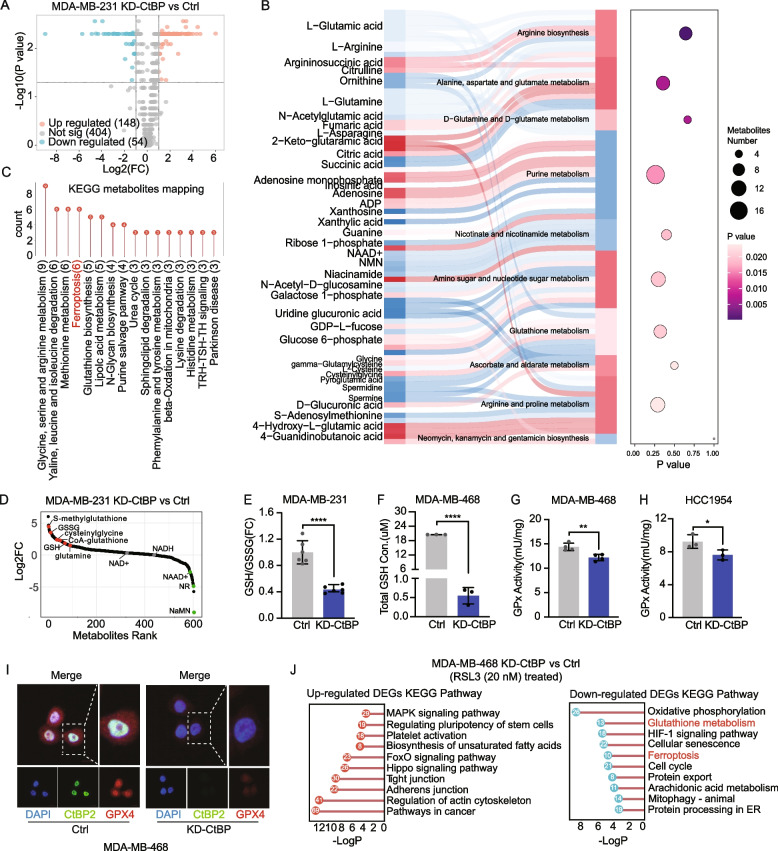
**A** Volcano plot of metabolomic data in MDA-MB-231 cells with CtBP KD. Blue and orange dots represent DPMs with |Log2FC|> 1 and an adjusted p-value < 0.05 (*n* = 6). **B** Lollipop chart of DPMs mapped to KEGG pathways. **C** Sankey diagram showing the top 10 KEGG metabolic pathways altered upon CtBP KD in MDA-MB-231 cells. Representative metabolites are shown on the left, while the number of enriched metabolites is displayed as bubbles on the right. **D** Ranking of altered metabolites in MDA-MB-231 cells upon CtBP KD. **E** Histogram showing the GSSG-to-GSH ratio, derived from the metabolomic data in (A)(*n* = 6). **F** Quantification of total glutathione in MDA-MB-468 cells with CtBP KD (*n* = 3). **G**, **H** Quantification of total glutathione peroxidase (GPx) activity in MDA-MB-468 (*n* = 4) and HCC1954 (*n* = 3) cells with CtBP KD. **I** IF staining of GPX4 (red), CtBP2 (green), and DAPI (blue) in MDA-MB-468 cells with CtBP KD. **J** KEGG pathway analysis of upregulated (left) and downregulated (right) differentially expressed genes (DEGs) (|Log2FC|> 0.5 and adjusted *p*-value < 0.05; *n* = 2) from RNA-Seq analysis of MDA-MB-468 cells with CtBP KD and treatment with 20 nM RSL3. For panels (**E**), (**F**), (**G**), and (**H**), *p*-values were calculated using a two-tailed Student’s t-test. #, *p* > 0.05; *, *p* < 0.05; **, *p* < 0.01; ***, *p* < 0.001; ****, *p* < 0.0001. Data are presented as mean ± SD (n ≥ 3)

Next, we performed targeted metabolomics analysis in MDA-MB-231 cells with over 600 potential targets semi-quantitated by normalized to total cell numbers (Fig. 2A). We found that the top differentially presented metabolites (DPMs) mainly belong to several metabolic pathways including Glutamine metabolism, Nicotinamide metabolism and glutathione metabolism etc. (Fig. 2B), which is partially consistent to our previous observation that CtBP regulates glutaminolysis pathway [24, 35]. KEGG pathway mapping identified ferroptosis as one of the most affected pathways upon CtBP KD (Fig. 2C). The ranking of these DPMs according to the fold change revealed that GSSG, the oxidized form of Glutathione showed a nearly 15-fold of increase, alongside other glutathione derivatives in MDA-MB-231 cell (Fig. 2D and sFig. 2G). GSH/GSSG ratio in CtBP KD cells decreased from 1.0 to 0.4 (fold change, p = 0.00002) (Fig. 2E), indicating a shift toward an oxidized intracellular state. Since the fold change of GSH/GSSG ratio was based on the data retrieved from the semi-quantitative metabolomics analysis which has limitation to reveal the true GSH/GSSG ratio, we further applied quantitative GSH kit to show that GSH decreased ~ 40-fold in MDA-MB-468 cells (Fig. 2F), suggesting CtBP is essential for maintaining the cellular GSH pool. Supporting this, total glutathione peroxidase (GPx) activity was significantly reduced in CtBP KD MDA-MB-468 and HCC1954 cells (Fig. 2G–H), and GPX4 protein levels were markedly diminished in CtBP KD cells (Fig. 2I). In contrast, other ferroptosis regulators (ACSL4, DHODH, FSP1, DHFR, GCH1) remained unchanged at both mRNA and protein levels across three TNBC cell lines (sFig. 2H–J). Lipidomic profiling showed no significant changes in phospholipids, lysophospholipids, or free fatty acids upon CtBP KD (sFig. 2 K). This result support CtBP inhibits ferroptosis via an antioxidant-centric mechanism rather than limiting the lipids peroxidation substrates.

To further assess the change of intracellular redox status upon CtBP KD, we performed RNA-seq analysis in MDA-MB-468 subjected to RSL3 induced ferroptosis. The KEGG analysis and GSEA analysis revealed that loss of CtBP resulted in dramatic changes of pathways such as glutathione metabolism, ferroptosis as well as ROS related pathways (Fig. 2J, sFig. 2L-2N). Consistently, a global reduction of antioxidant genes expression upon CtBP KD in all three TNBC cell lines could be further confirmed (sFig. 2O). Conclusively, the data provides compelling evidence to support CtBP function in overcoming oxidative stress and ferroptosis by upregulating glutathione pathway.

### CtBP regulates ferroptosis response in TNBC

**Fig. 3:**
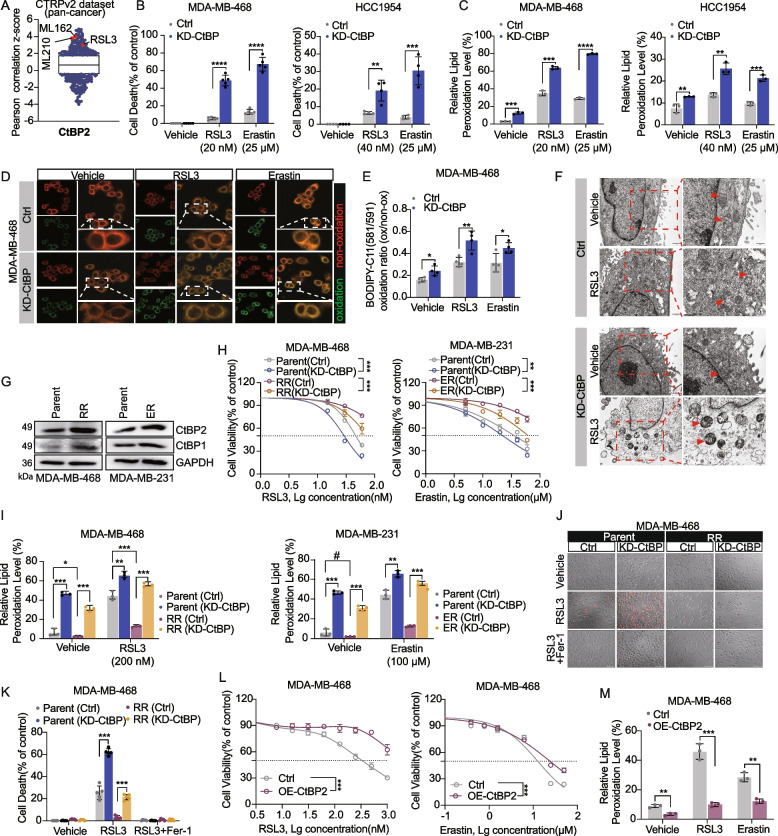
**A** Pearson correlation analysis between CtBP2 mRNA expression and drug sensitivity (shown as AUC, area-under-concentration-dependent-response curve) across cancer cell lines from the CTRP dataset. Red dots indicate ferroptosis inducers RSL3, ML210, and ML162. **B** Quantification of cell death by PI staining in cells with CtBP KD following treatment with RSL3 (20 nM, 40 nM) or Erastin (25 µM) for 24 h in MDA-MB-468 (*n* = 5) and HCC1954 (*n* = 4) cells. **C** Flow cytometry and (**D**) representative fluorescence microscopy images of BODIPY-C11 staining in MDA-MB-468 cells with CtBP KD, treated with RSL3 (20 nM) or Erastin (25 µM) (*n* = 3). Red indicates non-oxidized lipids; green indicates oxidized lipids. **E** Quantification of lipid peroxidation as the ratio of oxidized to non-oxidized lipids from (**D**) (*n* = 4). **F** Transmission electron microscopy (TEM) images showing mitochondrial morphology in MDA-MB-468 cells with CtBP KD treated with RSL3 (20 nM). (**G**) Western blot analysis of CtBP expression in RSL3-resistant (RR) MDA-MB-468 and Erastin-resistant (ER) MDA-MB-231 cells compared to parental controls (*n* = 3). **H** Cell viability and (I) lipid peroxidation in RR and ER cells and their parental counterparts treated with RSL3 (200 nM) or Erastin (100 µM) for 24 h (*n* = 3). **J** Representative PI staining images of cell death in RR and parental MDA-MB-468 cells treated with RSL3 (200 nM), with or without Ferrostatin-1 (2 µM). **K** Quantification of cell death from (J) (*n* = 5). **L** Cell viability and (M) lipid peroxidation in MDA-MB-468 cells overexpressing CtBP2 following treatment with RSL3 (20 nM) or Erastin (25 µM) (*n* = 3). For (B), (C), (E), (H), (I), (K), (L), and (M), statistical significance was determined using a two-tailed Student’s t-test. Data are presented as mean ± s.d. (n ≥ 3). #, *p* > 0.05; *, *p* < 0.05; **, *p* < 0.01; ***, *p* < 0.001; ****, *p* < 0.0001

CtBP2 expression is also positively correlated with the resistance to several ferroptosis inducers in the pan-cancer dataset [36] (Fig. 3A). Among the five BC cell lines, we noticed that TNBC cell lines show a more significant correlation between RSL3/Erastin induction and lipids peroxidation/cell death, suggesting TNBC cells are more sensitive to ferroptosis induction (sFig. 3 A). Therefore, we choose MDA-MB-468 and HCC1954 for further validation of CtBP regulation of ferroptosis activity.

As expected, both cells show increased oxidative stress and cell death in response to RSL3 or Erastin induction upon CtBP KD (Fig. 3B and sFig. 3B). Consistently, the lipids peroxidation is also increased significantly by CtBP KD in response to RSL3 and Erastin induction (Fig. 3C). In MDA-MB-468 cells, CtBP KD also resulted in a significant increased staining of peroxidized lipids upon treatment by RSL3 or Erastin (Fig. 3D and E). Moreover, the condensed mitochondria were more abundant in cells than the control cells while these cells were induced for ferroptosis by RSL3 (Fig. 3F). Next, we exposed MDA-MB-468 or MDA-MB-231 cells to continuous RSL3 (200 nM) or Erastin (100 µM) treatment respectively to select a RSL3 resistant cell line (RR) and Erastin resistant cell line (ER). Both RR and ER cells show increased expression of CtBP, along with a significant increase of oxidative stress (Fig. 3G, sFig. 3 C and sFig. 3D). In these RR and ER cells, CtBP KD still resulted in a significant increase of cell death and lipid peroxidation in response to RSL3/Erastin treatment, although the effect is not as dramatic as in the parental cells (sFig. 3E and sFig. 3 F). Moreover, knocking down of CtBP in RR or ER cells resulted in decreased cell viability (Fig. 3H and sFig. 3G) and increased lipids peroxidation (Fig. 3I and sFig. 3G) in response to RSL3 or Erastin treatment, with RR and ER cells showing more dramatic resistance. Consistently, we also observed the repression of cell death in RSL3 treated RR cells with CtBP KD (Fig. 3J, K). In addition, ectopic expression of CtBP2 in MDA-MB-468 cells significantly increased the cell resistance to RSL3 or Erastin resistance (Fig. 3L, M and sFig. 3H). Together, these data indicate CtBP contributes to the ferroptosis resistance in TNBC.

### P4HB mediates CtBP function in regulating ferroptosis

**Fig. 4:**
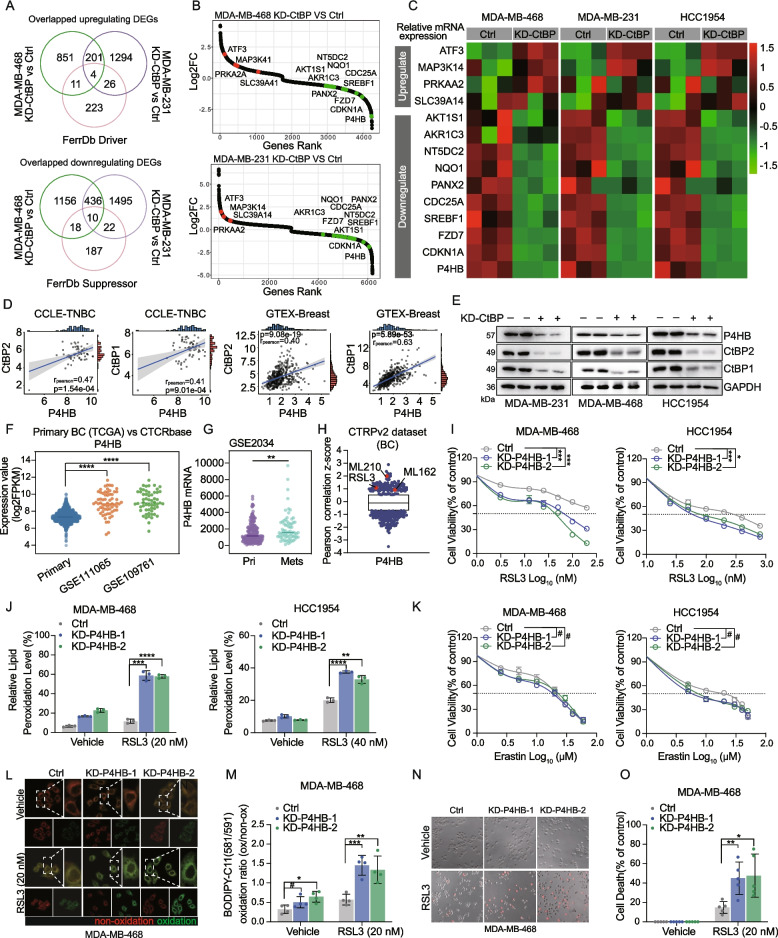
**A** Venn diagrams showing overlap between DEGs (|log₂FC|> 0.5, adjusted *P* < 0.05) upon CtBP KD in MDA-MB-231 and MDA-MB-468 cells and ferroptosis-related genes from FerrDb. Top: upregulated DEGs overlapping with ferroptosis drivers; bottom: downregulated DEGs overlapping with ferroptosis repressors. **B** Ranked DEGs upon CtBP KD in MDA-MB-468 (top) and MDA-MB-231 (bottom). Red and green dots indicate ferroptosis drivers and repressors, respectively. **C** Heatmap showing RT–PCR fold changes of 14 candidate genes upon CtBP KD in MDA-MB-468, MDA-MB-231, and HCC1954 cells (*n* = 3). **D** Pearson correlation between P4HB and CtBP1 or CtBP2 mRNA expression in TNBC cell lines (CCLE dataset) (*n* = 36) and normal mammary tissue samples (GTEx dataset) (*n* = 459). **E** Western blot quantification of P4HB, CtBP1, and CtBP2 in MDA-MB-231, MDA-MB-468, and HCC1954 cells with CtBP KD (*n* = 3). **F** Comparison of P4HB mRNA expression (FPKM) between primary breast tumors (TCGA-BRCA, *n* = 1217) and circulating tumor cells (ctcRbase) (GSE111065 *n* = 65, GSE109761 *n* = 57). **G** Comparison of P4HB mRNA levels in matched primary (*n* = 217) and metastatic breast tumors (GSE2034, *n* = 69). **H** Pearson correlation between P4HB mRNA expression and drug sensitivity (AUC) across cancer cell lines from the CTRP dataset. Red dots indicate ferroptosis inducers RSL3, ML210, and ML162. **I** Cell viability of MDA-MB-468 and HCC1954 cells with P4HB KD following RSL3 treatment (*n* = 3). **J** Flow cytometry analysis of lipid peroxidation using BODIPY-C11 staining in MDA-MB-468 and HCC1954 cells treated with RSL3 (20 nM and 40 nM, respectively) for 24 h (*n* = 3). **K** Cell viability of MDA-MB-468 and HCC1954 cells with P4HB KD following Erastin treatment (*n* = 3). **L** Representative fluorescence microscopy images of BODIPY-C11 staining in MDA-MB-468 cells with P4HB KD treated with RSL3 (20 nM). Red: non-oxidized lipids; green: oxidized lipids. **M** Quantification of oxidized-to-non-oxidized lipid ratio from (L) (*n* = 4). **N** Representative PI staining images showing cell death in MDA-MB-468 cells with P4HB KD treated with RSL3 (20 nM). **O** Quantification of cell death from (N) (*n* = 5). For (D) and (H), data were analyzed using Pearson correlation. For (C), (I), (J), (K), (M), and (O), statistical significance was determined using a two-tailed Student’s t-test. Data are presented as mean ± s.d. (n ≥ 3). #, *p* > 0.05; *, *p* < 0.05; **, *p* < 0.01; ***, *p* < 0.001; ****, *p* < 0.0001. For (F) and (G), data are shown as median with interquartile range

The known function of CtBP is regulating chromatin accessibility and gene transcription. We identified 4 up-regulated and 10 down-regulated DEGs (differentially expressed genes) by overlapping the DEGS from MDA-MB-231 and MDA-MB-468 cells upon CtBP KD and the FerrDb Drivers or Suppressors [37] (Fig. 4A and B). We validated the change of these candidate genes expression upon CtBP KD in three TNBC cell lines and identified P4HB as a candidate gene in mediating CtBP function in increasing ferroptosis resistance (Fig. 4C, sFig. 4A-4C). To our interest, P4HB expression is significantly upregulated in breast cancer in comparison with the normal mammary gland tissue (sFig. 4D-4E). A previously published dataset also showed P4HB downregulation upon CtBP KD (sFig. 4 F). The positive correlation between CtBP expression and P4HB expression was also observed in both CCLE-TNBC cells and GTEX-breast cancer dataset (Fig. 4D). Interestingly, only P4HB mRNA shows the most dramatic decrease upon CtBP KD in all three TNBC cells but not the other PDI family genes (sFig. 4G). Consistently, decreased expression of P4HB at protein level upon CtBP KD was further confirmed in TNBC cells (Fig. 4E and sFig. 4H). Comparing to the primary tumor, P4HB is significantly upregulated in circulating tumor cells (Fig. 4F). Moreover, a subtle but significant higher expression of P4HB is observed in metastatic tumors compared to primary tumors (Fig. 4G). Like CtBP2, P4HB expression is also positively correlated with the resistance to several ferroptosis inducers in the CTRPv2 dataset [36] (Fig. 4H). To understand the mechanism through which CtBP regulates P4HB gene expression, we identified a CtBP binding peak at P4HB promoter based on CtBP ChIP-seq data. ChIP-qPCR analyses could validate this binding peak as expected. Dual-luciferase reporter assays further demonstrated that CtBP KD suppressed P4HB promoter activity by ~ 50% while CtBP2 overexpression significantly increased P4HB mRNA transcription. Collectively, these data established P4HB as a direct transcriptional target of CTBP2 (sFig. 4I-J). Next, we studied if P4HB is a regulator of ferroptosis in TNBC. As we expected, P4HB KD resulted in the reduction of cell viability and increased lipid peroxidation in response to RSL3 treatment (Fig. 4I and J, sFig. 4 K). But both TNBCs showed less responsiveness to Erastin treatment upon P4HB KD (Fig. 4K). Upon P4HB KD, the Bodipy-C11—581/591 staining revealed an increased lipids peroxidation and cell death in response to RSL3 treatment (Fig. 4L-O). Together, these data indicated an essential role of P4HB in protecting TNBC cells from ferroptosis.

### Essentialness of Endoplasmic reticulum localization and disulfide-isomerase activity of P4HB in contributing to ferroptosis resistance

**Fig. 5:**
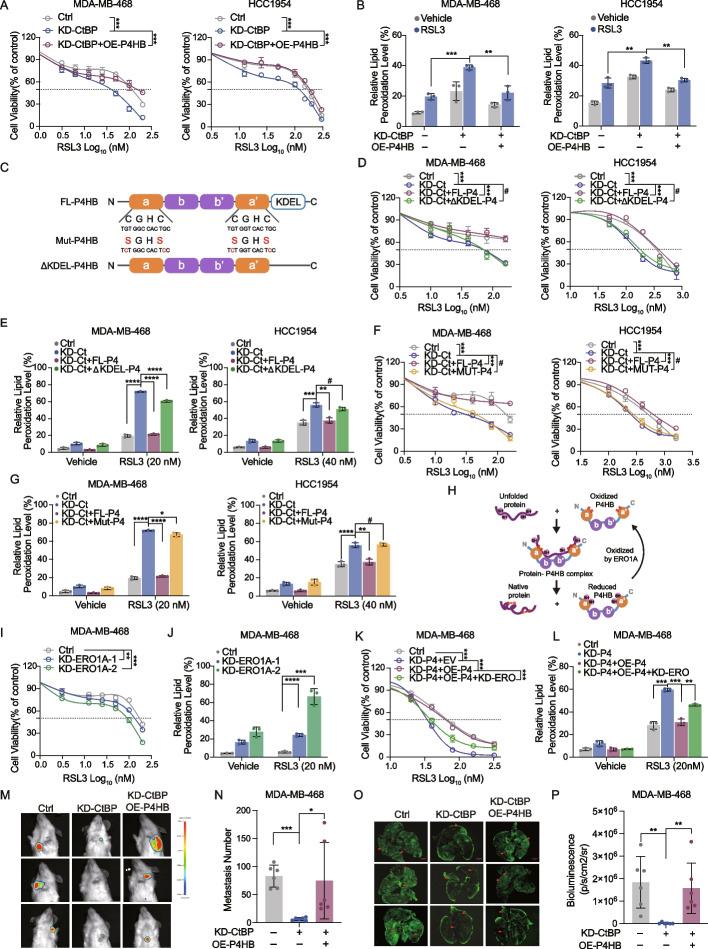
**A** Cell viability and (**B**) lipid peroxidation in MDA-MB-468 and HCC1954 cells treated with RSL3 under the indicated conditions (*n* = 3). **C** Schematic representation of P4HB structure and mutation design. Mut-P4HB indicates cysteine-to-serine substitutions; ΔKDEL-P4HB denotes deletion of the ER retention motif. **D**, **F** Cell viability and (**E**, **G**) lipid peroxidation in MDA-MB-468 and HCC1954 cells expressing wild-type or mutant P4HB constructs and treated with RSL3 (*n* = 3). (H) Schematic illustrating the oxidative folding of nascent proteins by P4HB via disulfide bond formation. P4HB oxidation is mediated by ERO1A. **I**, **K** Cell viability and (**J**, **L**) lipid peroxidation in MDA-MB-468 cells with indicated conditions and RSL3 treatment (*n* = 3). **N**–**Q** Lung metastasis model using tail vein injection of 2 × 10⁶ luciferase- and GFP-labeled MDA-MB-468 cells into NSG mice (*n* = 6). (N) Bioluminescence imaging and (O) quantification of metastatic burden. **P** Representative fluorescence images showing GFP-positive metastatic foci in lung tissue (*n* = 6). **Q** Quantification of metastatic events. For (A), (B), (D), (E), (F), (G), (I), (J), (K), (L), (O), and (Q), statistical significance was determined using a two-tailed Student’s t-test. Data are presented as mean ± s.d. (n ≥ 3).). #, *p* > 0.05; *, *p* < 0.05; **, *p* < 0.01; ***, *p* < 0.001; ****, *p* < 0.0001

To investigate how P4HB, as the mediator of CtBP, contributes to the upregulation of GPX4-GSH pathway, we ectopically expressed P4HB in TNBC cells. As expected, P4HB significantly decreased ROS in CtBP KD TNBC cells and rescued the cell from RSL3 induced ferroptosis (Fig. 5A and B, sFig. 5 A and sFig. 5B). P4HB belongs to protein disulfide isomerase (PDIs) family protein and plays an important role in protein folding during maturation in ER. The P4HB protein is 55 kDa, and contains four thioredoxin domains, designated as a, b, a', and b', as well as a C-terminal domain. The a and a' domains contain the CGHC motif, which serves as the reaction center of thioredoxin domains. The b and b' domains are catalytically inactive but responsible for substrate binding and chaperone function. P4HB is not a ferroptosis related factor but a recent study identified a ferroptosis related circle RNA at P4HB gene locus [38] (Fig. 5C). By mutating the two CGHC motifs, we generated Mut-P4HB which loss the isomerase activity. Simultaneously, we also generated ΔKDEL-P4HB mutant which loss the ER localization signal (Fig. 5C, sFig. 5 C and sFig. 5D). Upon RSL3 treatment, while the FL-P4HB (full length-P4HB) could rescue the cells from CtBP KD induced ferroptosis, both Mut-P4HB or KDEL-P4HB mutants failed to rescue the cells from ferroptosis, suggesting the anti-ferroptosis activity of P4HB requires its isomerase activity and its ER localization (Fig. 5D-G). ERO1A is a known regulator of P4HB which is essential to recover the non-oxidized form of P4HB (Fig. 5H). ERO1A KD in RSL3 treated cells resulted in increased ferroptosis (Fig. 5I, J and sFig. 5E). Moreover, we observed that ERO1A is essential to support the P4HB function in repressing RSL3 induced ferroptosis (Fig. 5K, L, and sFig. 5 F). Finally, we verified that P4HB overexpression promotes the lung metastasis of MDA-MB-468 cells with CtBP KD using the tail vein injection model (Fig. 5N-Q and sFig. 5G). Together, these data support that P4HB mediates CtBP function in promoting ferroptosis resistance and TNBC metastasis.

### P4HB increases SLC7A11 maturation and membrane localization

**Fig. 6:**
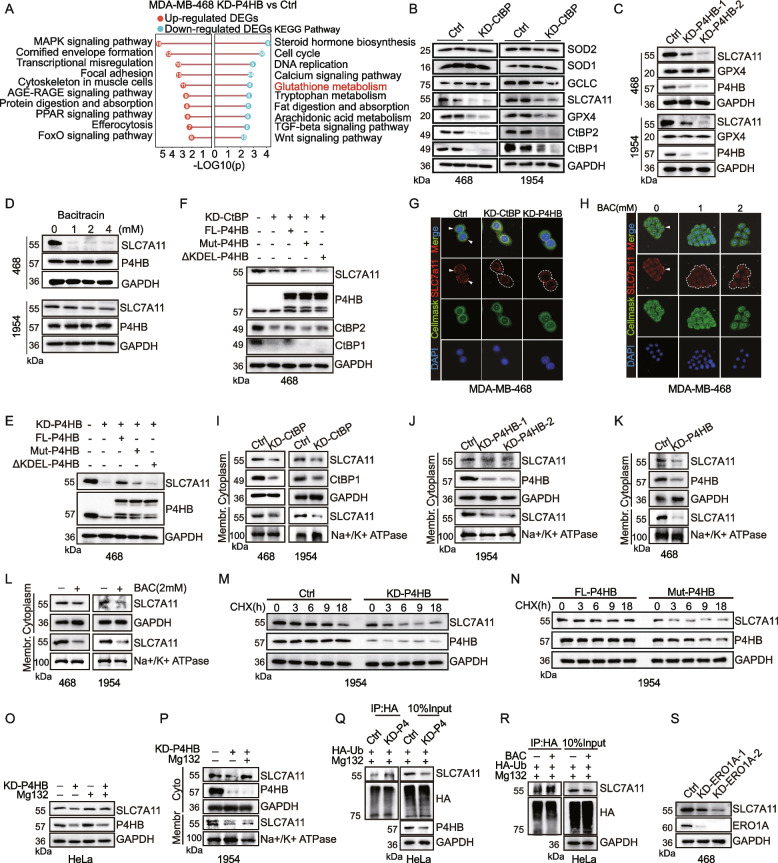
**A** KEGG pathway enrichment analysis of DEGs in MDA-MB-468 cells following P4HB knockdown (|log₂FC|> 0.5, adjusted *P* < 0.05; *n* = 2). **B** WB quantification of indicated proteins in MDA-MB-468 and HCC1954 cells with CtBP KD (*n* = 3). **C**, **D** WB quantification of indicated proteins in MDA-MB-468 and HCC1954 cells with P4HB KD (C) or treated with the P4HB inhibitor bacitracin (0–4 mM) for 24 h (D) (*n* = 3). **E**, **F** WB quantification of SLC7A11 and P4HB in MDA-MB-468 cells with P4HB KD (**E**) or CtBP KD (F), in combination with overexpression of wild-type or mutant P4HB (*n* = 3). **G**, **H** IF staining of SLC7A11 (red), CellMask (green), and DAPI (blue) in MDA-MB-468 cells with CtBP and P4HB KD (**G**), or treated with bacitracin at indicated concentrations for 24 h (**H**). **I**–**K** WB quantification of membrane and cytosolic fractions of indicated proteins in MDA-MB-468 and HCC1954 cells with CtBP KD (**I**) or P4HB KD (**J**, **K**) (*n* = 3). **L** WB quantification of membrane and cytosolic proteins in MDA-MB-468 and HCC1954 cells treated with bacitracin (2 mM) for 24 h (*n* = 3). (M, N) WB quantification of SLC7A11 and P4HB in HCC1954 cells treated with cycloheximide (CHX, 30 µg/mL) for indicated durations, with P4HB KD (M) or overexpression of wild-type or mutant P4HB (N) (*n* = 3). **O** WB quantification of SLC7A11 and P4HB in HeLa cells with P4HB knockdown and MG132 (20 µM) treatment for 8 h (*n* = 3). **P** WB quantification of membrane and cytosolic proteins in HCC1954 cells treated with MG132 (20 µM) for 8 h (*n* = 3). **Q**, **R** WB quantification of ubiquitinated SLC7A11 (HA-Ub modified) in HeLa cells with P4HB knockdown (**Q**) or bacitracin (2 mM) treatment for 24 h (R), in the presence of MG132 (20 µM) (*n* = 3). **S** WB quantification of SLC7A11 following ERO1A knockdown in MDA-MB-468 cells (*n* = 3). For (A), statistical significance was determined using a two-tailed Student’s t-test. Data are presented as mean ± s.d. (n ≥ 3). #, *p* > 0.05; *, *p* < 0.05; **, *p* < 0.01; ***, *p* < 0.001; ****, *p* < 0.0001

P4HB high expressed tumors show enrichment of glutathione metabolism in TCGA-BC dataset (sFig. 6 A). The glutathione metabolism function is also the top enriched KEGG term by analyzing the DEGs derived from P4HB KD MDA-MB-468 cells (Fig. 6A and sFig. 6B), suggesting P4HB might play important role in promoting the antioxidant capacity which is critical to prevent ferroptosis. In CtBP KD TNBC cells, we observed a uniform reduction of antioxidant genes (sFig. 6 C). Meanwhile, both GPX4 and SLC7A11 proteins, but not other antioxidant genes, show significant decrease in CtBP KD TNBC cells (Fig. 6B and sFig. 6D). Since P4HB plays a critical role in protein folding, we further examined if GPX4 and SLC7A11 post-translational folding requires P4HB. In P4HB KD cells, only SLC7A11 decreases significantly but not GPX4 (Fig. 6C). Bacitracin is a known inhibitor of P4HB, and bacitracin treatment resulted in a significant decrease of SLC7A11 (Fig. 6D), suggesting SLC7A11 is a candidate target protein of P4HB disulfide isomerase activity. SLC7A11 mediates the exchange of extracellular cystine and intracellular glutamate and plays a critical role to maintain intracellular cystine, and thereafter the glutathione synthesis. Consistently, a positive correlation between SLC7A11 and CtBP was observed (sFig. 6E and sFig. 6 F). Interestingly, P4HB KD or inhibition also leads to a significant increase in SLC7A11 mRNA expression (sFig. 6G and sFig. 6H), probably owing to an unknown feedback mechanism. Next, we further confirmed that FL-P4HB, but not Mut-P4HB or ΔKDEL-P4HB, could recover SLC7A11 expression in P4HB KD cells or CtBP KD cells (Fig. 6E and F). Under fluorescence confocal microscope, we noticed that the membrane localization of SLC7A11 is greatly reduced at cell membrane but enriched at paranuclear region (where ER localized) upon CtBP KD or P4HB KD (Fig. 6G). P4HB inhibition by bacitracin also abolished the membrane localization of SLC7A11 (Fig. 6H). Consistently, membrane located SLC7A11 is significantly reduced upon CtBP KD, P4HB KD, or P4HB inhibition (Fig. 6I-L and sFig. 6I-K), suggesting P4HB is essential for SLC7A11 membrane localization. Intriguingly, the cytosolic SLC7A11 is also decreased by CtBP KD, P4HB KD or P4HB inhibition (Fig. 6I-L and sFig. 6I-K). We hypothesized that P4HB KD or inhibition cause SLC7A11 degradation owing to misfolding. As we expected, we observed that P4HB KD results in the accelerated degradation of SLC7A11 upon CHX treatment (Fig. 6M). Moreover, mut-P4HB shows reduced ability to maintain SLC7A11 expression when the de novo synthesis of SLC7A11 was blocked by CHX (Fig. 6N). Instead, MG132 treatment successfully recovered SLC7A11 upon P4HB KD (Fig. 6O). But this recovery only limited to cytoplasmic SLC7A11 but not the membrane associated SLC7A11, suggesting MG132 only prohibited the SLC7A11 degradation but didn’t recover SLC7A11 from misfolding (Fig. 6P). Next, we further validated that ubiquitinated SLC7A11 is increased upon P4HB KD or P4HB being inhibited by BAC, among all the ubiquitinated protein (Fig. 6Q and R). Co-IP analysis validated the physical interaction between P4HB and SLC7A11 (sFig. 6L), and functional assays demonstrated that SLC7A11-mediated cystine transport acts downstream of P4HB: exogenous wild-type SLC7A11 failed to restore cystine uptake in P4HB-deficient cells, whereas the C158-deficient mutant (disrupting SLC3A2 heterodimerization) significantly attenuated cystine uptake in P4HB-overexpressing cells compared to wild-type (sFig. 6 M). Consistently, ERO1A KD resulted in the decreased SLC7A11 as expected (Fig. 6S). Together, our data revealed P4HB dependent SLC7A11 protein folding and membrane localization which is essential to mediate the CtBP function in promoting the intracellular antioxidant and ferroptosis resistance.

### Ferroptosis resistance by CtBP-P4HB-SLC7A11 axis is a therapeutic target in breast cancer treatment

**Fig. 7:**
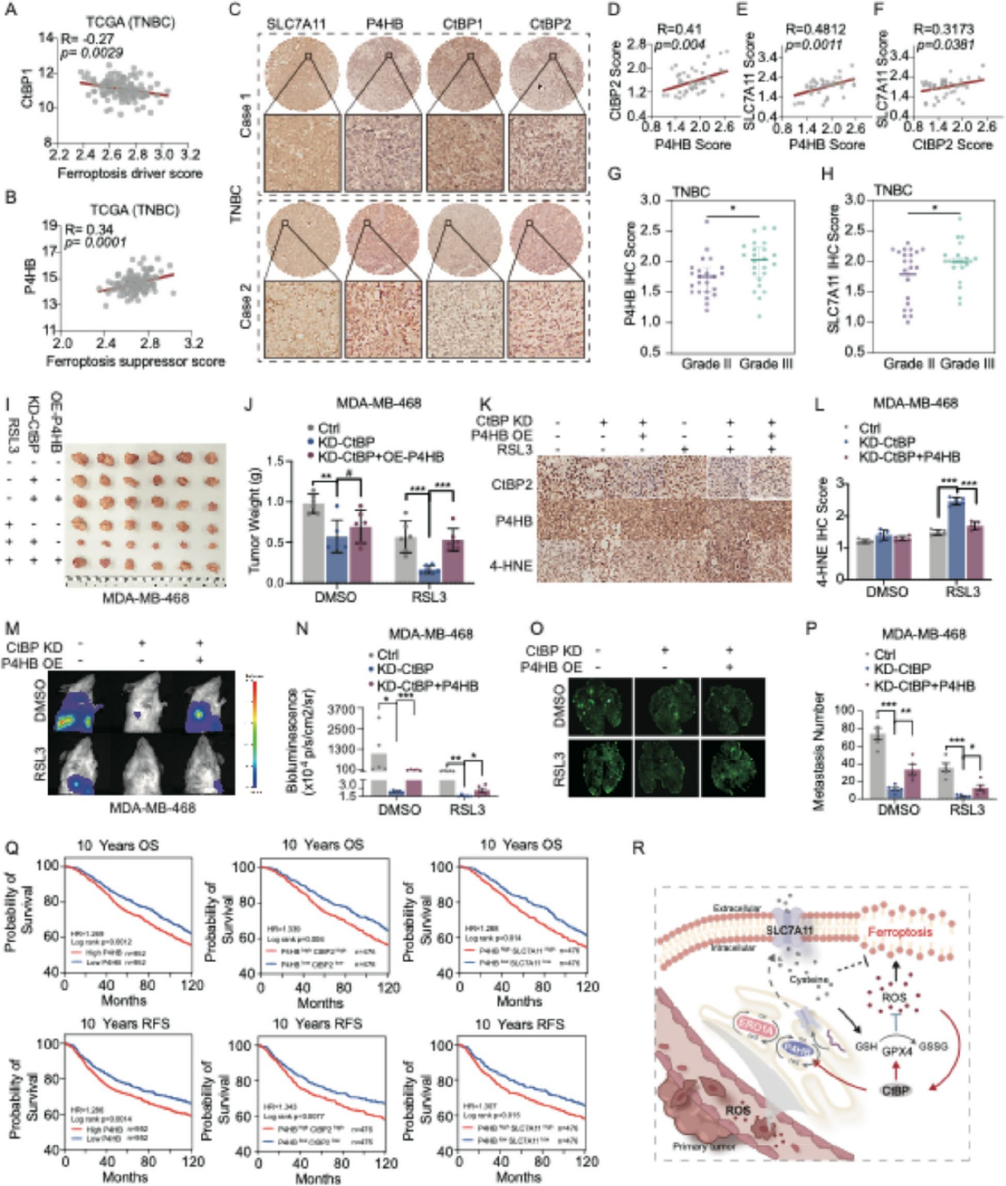
(**A**, **B**) Pearson correlation between CtBP1 mRNA and ferroptosis driver score (**A**), and between P4HB mRNA and ferroptosis suppressor score (**B**) in TCGA triple-negative breast cancer (TNBC) samples (*n* = 123). Ferroptosis-related gene sets were curated from FerrDb and scores were calculated using single-sample ssGSEA. **C** Representative IHC images of SLC7A11, P4HB, CtBP1, and CtBP2 in 48 TNBC tumor samples. **D**–**F** Pearson correlation between IHC scores of P4HB and CtBP2 (**D**), P4HB and SLC7A11 (**E**), and CtBP2 and SLC7A11 (**F**) in TNBC samples (*n* = 48). **G**, **H** Scatter plots showing IHC scores of P4HB (**G**) and SLC7A11 (**H**) stratified by tumor grade in TNBC samples (*n* = 48). **I** Xenograft tumors generated by subcutaneous injection of 5 × 10⁶ MDA-MB-468 cells under indicated conditions, followed by intra-tumoral injection of RSL3 (50 mg/kg) twice weekly for two weeks (*n* = 6). **J** Quantification of tumor weight following treatment. **K** IHC staining of CtBP2, P4HB, and 4-HNE in tumor sections. **L** Quantification of 4-HNE IHC scores from (**K**). **M**–**P** Lung metastasis model using tail vein injection of 2 × 10⁶ luciferase- and GFP-labeled MDA-MB-468 cells with CtBP KD, P4HB OE, or both, with or without RSL3 pre-treatment (20 nM, 24 h). Bioluminescence imaging and quantification are shown in (**M**) and (**N**); GFP-positive metastatic foci are shown in (**O**) and quantified in (**P**) (*n* = 5). **Q** Kaplan–Meier survival analysis of overall survival (OS) and recurrence-free survival (RFS) in breast cancer patients from the METABRIC cohort. Comparisons include P4HBhigh vs P4HBlow (*n* = 952), P4HBhighCtBPhigh vs P4HBlowCtBPlow (*n* = 476), and P4HBhighSLC7A11high vs P4HBlowSLC7A11low (*n* = 476). **R** Proposed working model: CtBP promotes ferroptosis resistance by enhancing P4HB-mediated maturation of SLC7A11 protein. For (G), (H), (J), (L), (N), and (P), statistical significance was determined using a two-tailed Student’s t-test. Data are presented as mean ± s.d. (n ≥ 3). For (Q), P values were calculated using a log-rank test. #, *p* > 0.05; *, *p* < 0.05; **, *p* < 0.01; ***, *p* < 0.001; ****, *p* < 0.0001

In TCGA-BC and METABRIC-TNBC datasets, CtBP showed a negative correlation to Ferroptosis driver score (Fig. 7A and sFig. 7 A). Meanwhile, P4HB showed a positive correlation to Ferroptosis suppressor score (Fig. 7B, sFig. 7B and sFig. 7 C), confirming the importance of CtBP-P4HB-SLC7A11 axis in promoting ferroptosis resistance. Next, we detected the correlation of CtBP, P4HB and SLC7A11 in 48 clinical TNBC samples by IHC (immunohistochemistry) (Fig. 7C). The positive correlation between CtBP and P4HB can be confirmed (CtBP1, r = 0.41, *p* = 0.004; CtBP2, r = 0.36, *p* = 0.01) (Fig. 7D). The positive correlation between SLC7A11 and P4HB is also significant (r = 0.48, *p* = 0.001) (Fig. 7E). We further observed the positive correlation between CtBP2 and SLC7A11 (r = 0.32, *p* = 0.04) (Fig. 7F). Importantly, higher P4HB expression and SLC7A11 expression are associated with more grade III than with grade II TNBC tumors (Fig. 7G and H), suggesting CtBP-P4HB-SLC7A11 axis likely attributes to higher grade TNBC.

To confirm P4HB is critical in mediating CtBP function in counteracting ferroptosis, we established xenografted tumor model using MDA-MB-468 cells with CtBP KD or CtBP KD combined with P4HB overexpression. Upon RSL3 treatment, we observed the significant retardation of tumor growth in CtBP KD group. P4HB overexpression recovered the tumor growth significantly (Fig. 7I and J). Consistently, the significant upregulation of ferroptosis as indicated by 4-HNE staining in CtBP KD group can be observed and P4HB overexpression significantly decreases ferroptosis in CtBP KD samples (Fig. 7K and L). These data suggest CtBP-P4HB axis is a critical pathway to promote the ferroptosis resistance. We also applied tail vein injection of MDA-MB-468 cells to observe metastatic colonies formation in lungs. Consistently, the metastatic foci number was greatly reduced by CtBP KD and was rescued by P4HB overexpression (Fig. 7M, N, O and P). RSL3 treatment potentiated the inhibitory effect of CtBP KD on metastasis and partially influenced the rescue effect of P4HB overexpression (Fig. 7M, N, O and P). Finally, we separated the METBRIC clinical samples into two groups according to the expression of P4HB alone, P4HB and CtBP in combination, or P4HB and SLC7A11 in combination respectively and compared the 10-year Overall Survival (OS) and the 10-year Recurrence-Free Survival (RFS) among different groups. In support of the importance of CtBP-P4HB-SLC7A11 axis in promoting cancer metastasis, P4HB^high^, P4HB^high^&CtBP2^high^, P4HB^high^&SLC7A11^high^ groups show significantly worse OS and RFS (Fig. 7Q). Together, the in vivo data supports CtBP-P4HB-SLC7A11 forms a novel pathway to promote intracellular antioxidant, mainly glutathione, and increase the cancer cell resistance to ferroptosis (Fig. 7R).

## Discussion

The emergence and progression of tumour cells is owing to the interplay between intrinsic and extrinsic factors. The tissue microenvironment represents the extrinsic force acting on cancer cells, whereas mutations and the rewiring of gene expression constitute the intrinsic factors that contribute to tumor development. The goal of such gene expression reprogramming is to enable cancer cells to adapt to their microenvironment and to acquire the capacity to migrate and survive in distant tissues. For instance, the rapid proliferation and metastatic potential of cancer cells expose them to elevated oxidative stress; accordingly, the acquisition of antioxidant defenses becomes essential for evading apoptosis [39, 40]. In this study, we demonstrate that the upregulation of CtBP expression in response to oxidative stress was observed in several cancer cell models when the cells are under experimental conditions mimicking the diverse stresses cancer cells encounter within the tissue microenvironment. Given that CtBP functions as a broadly acting transcriptional cofactor [13], its induction under oxidative stresses suggests a role in the adaptive response to these stresses. Importantly, CtBP expression exhibits strong adaptability to oxidative conditions since its expression returns to baseline levels upon stress removal.

Under oxidative stresses, several cell death pathways will be activated. High expression of CtBP associates with the resistance of these cell death pathways such as apoptosis, autophagy etc. While the mechanisms through which CtBP promotes the apoptosis resistance in several types of cancer have been demonstrated, CtBP increases the ferroptosis resistance in TNBC is a novel finding in this research. In our observation, when ferroptosis inducing chemicals were applied, CtBP expression is also upregulated because these chemicals induce ferroptosis through creating oxidative stress. Meanwhile, we further demonstrated that the cells chronically exposed to ferroptosis inducer RSL3 rely on CtBP to establish the ferroptosis resistance (for example, the RR cells in Fig. 3H). However, CtBP KD in RR cells only mildly increased the sensitivity to RSL3 induction, suggesting chronically exposure of the cells to RSL3 not only upregulates CtBP but also motivates other mechanisms to form ferroptosis resistance.

Mechanistically, we identified P4HB as a mediator through which CtBP promotes ferroptosis resistance. Interestingly, although P4HB was identified from FerrDB, this discovery seems owing to a serendipity because P4HB was included in FerrDB not because previous studies had established a direct link to ferroptosis, but because a circular RNA (circRNA) derived from the P4HB gene locus had been reported to be associated with ferroptosis [38]. Fortunately, our experimental results confirm that P4HB enhances ferroptosis resistance in triple-negative breast cancer (TNBC). Thus, the fact that the P4HB locus produces a ferroptosis-regulatory circRNA appears non-random, although the potential relationship between these two mechanisms remains unclear.

While CtBP has occasionally been reported to promote the expression of certain genes through transcriptional regulation [41], it more commonly functions as a transcriptional repressor. We haven’t attempted to explore the mechanism by which CtBP enhances P4HB expression yet. However, we observed that P4HB protein level decreases upon CtBP KD, confirming that P4HB expression responds to CtBP, despite the lack of a defined regulatory pathway. Indeed, in tissue microarrays, we also detected a positive correlation between the expression of P4HB and CtBP. But whether P4HB is a direct transactivation target of CtBP is still unknown.

Upon P4HB KD, we noticed that the cells become no response to Erastin treatment but still sensitive to RSL3 treatment (Fig. 4I-K). Once we identified SLC7A11 is regulated by P4HB, we understand that the cells become no response to Erastin because P4HB and Erastin share the same target protein SLC7A11. Upon P4HB KD, SLC7A11 has low presence on cell membrane and Erastin could no longer influence the intracellular redox status mediated by SLC7A11.

In our initial experiments, we were intrigued by the marked downregulation of GPX4 upon CtBP KD. As a central mediator of ferroptosis resistance, reduced GPX4 expression would be expected to sensitize cells to ferroptosis induction [42]. However, P4HB overexpression enhances ferroptosis resistance in CtBP-KD cells. In other words, P4HB rescues the cells from ferroptosis when GPX4 is downregulated by CtBP KD, suggesting CtBP protects cells from ferroptosis through at least two distinct pathways: one is CtBP-P4HB-SLC7A11, and the other is possibly CtBP-GPX4 pathway. However, how CtBP KD resulted in the reduced GPX4 expression is unknown. But P4HB rescue of ferroptosis resistance in CtBP KD cells in a GPX4 independent manner is possibly explained by the following reasons. For instance, while our data, combined with prior literature [43], support SLC7A11 as a downstream mediator of P4HB’s anti-ferroptosis activity, this mechanism lacks exclusivity. We cannot rule out additional P4HB substrates that may independently confer ferroptosis resistance—a scenario consistent with the widely recognized redundancy in ferroptosis regulation. Furthermore, while cysteine availability is traditionally associate with glutathione (GSH) biosynthesis—supporting GPX4-mediated suppression of ferroptosis—alternative GSH-dependent but GPX4-independent pathways remain incompletely characterized [44]. Notably, the glutathione peroxidase family includes at least 8 members, and GSH also provides reducing equivalents for processes beyond GPX4 function. Additionally, regenerating vitamin E (a critical lipid peroxidation scavenger) via vitamin C-dependent mechanisms (which requires glutathione as well) is also known to counteract ferroptosis [45]. In fact, P4HB overexpression only partially rescues cell viability in CtBP-KD cells. Lastly, GPX4 may not be the rate-limiting factor in ferroptosis resistance and decreased GPX4 expression does not equate to complete loss of its enzymatic activity [44]. Thus, exploring how the P4HB-SLC7A11 axis mediates ferroptosis resistance independent of GPX4 represents a promising avenue for further investigation.

MDA-MB-231 cell line is widely used as a TNBC model. Intriguingly, MDA-MB-231 cells only show sensitivity to Erastin-induced ferroptosis, with negligible response to RSL3 induced ferroptosis. Another intriguing observation is that metabolomic analyses of this cell line revealed concurrent increases in both GSH and GSSG upon CtBP depletion, although GSH/GSSG ration still decreases which agrees with the conclusion that CtBP KD exacerbates intracellular oxidative stress. Notably, MDA-MB-231 cells exhibit higher SLC7A11 protein expression than other TNBC cell lines (sFig. 6E). Given the extremely aggressive phenotype of this cell line, we speculate that MDA-MB-231 cells may adopt some mutations driving constitutive SLC7A11 overexpression—potentially preserving its expression even upon CtBP KD or P4HB KD. Such elevated SLC7A11 would maintain a large intracellular glutathione pool, which could explain why CtBP depletion fails to alter RSL3-induced ferroptosis. Additionally, the abundant glutathione might overcome ferroptosis induction via GPX4-independent mechanisms. For instance, abundant glutathione might overcome ferroptosis induction via GPX4-independent mechanisms. This aligns with our observations in other TNBC lines, where CtBP KD reduces GPX4 but also depletes glutathione, potentially disrupting additional antioxidant pathways beyond GPX4 (see discussion above).

TNBC remains a therapeutic challenge due to its pronounced intratumoral heterogeneity and inherent or acquired resistance to chemotherapy. The induction of ferroptosis—driven by elevated intracellular oxidative stress, iron accumulation, and lipid peroxidation—has thus emerged as an attractive therapeutic strategy. However, the decentralized and highly redundant nature of ferroptosis regulatory networks often leads to development of resistance to ferroptosis induction [46]. Therefore, the more we understand about the mechanisms underlying ferroptosis resistance, the better equipped we are to develop effective clinical strategies. This knowledge allows the simultaneous targeting of multiple pathways that frequently contribute to ferroptosis resistance. Furthermore, the identification of novel marker genes for ferroptosis resistance allows for patient selection and more precise, personalized therapeutic approaches. For example, our results suggest the distinguishment of a subset of TNBC with high CtBP expression that confers ferroptosis resistance by engaging the P4HB–SLC7A11 pathway. Notably, P4HB has previously been implicated in tumor progression through diverse mechanisms, and specific inhibitors are under development. Our findings provide new insights into the potential mechanisms of action of P4HB inhibitors and suggest their utility in combination with ferroptosis inducers for the treatment of TNBC.

## Conclusions

In summary, this study establishes the CtBP-P4HB-SLC7A11 axis as a pivotal determinant of ferroptosis sensitivity in triple-negative breast cancer. While SLC7A11 is a well-known guardian against ferroptosis, the upstream mechanisms regulating its maturation and membrane localization have remained elusive. Our data fill this gap by identifying the CtBP-dependent upregulation of P4HB as a prerequisite for functional system xc − activity. We show that without the chaperone activity of P4HB, SLC7A11 fails to reach the plasma membrane, leading to glutathione depletion and lipid peroxidation. Therefore, the high expression of these proteins observed in clinical TNBC samples not only serves as a potential prognostic biomarker but also highlights a specific therapeutic target. Disrupting this axis offers a novel approach to dismantle the antioxidant defenses of tumor cells and enhance the efficacy of ferroptosis-based treatments.

## Supplementary Information

Supplementary Material 1.

## Data Availability

All dataset are provided as supplementary material.

## Abbreviations

ACSL4: Acetyl-CoA synthase long chain family 4
CtBP: C-terminal Binding Protein
CTCs: Circulating tumor cells
CHX: Cycloheximide
CCLE: Cancer Cell Line Encyclopedia
CTRP: The Cancer Therapeutics Response Portal
DEGs: Differential expression genes
DPMs: Differentially presented metabolites
DMEM: Dulbecco's Modified Eagle Medium
ER: Endoplasmic reticulum
FSP1: Ferroptosis suppressor protein 1
FBS: Fetal Bovine Serum
GSH: Glutathione
GSSG: Oxidized glutathione
GPX: Glutathione peroxidase
GO analysis: Gene ontology analysis
IHC staining: Immunohistochemical staining
KD: Knockdown
KEGG: Kyoto Encyclopedia of Genes and Genomes
NSG mice: NOD scid gamma mice
NADPH: Reduced phosphorylated form of nicotinamide adenine dinucleotide
OE: Overexpression
PUFAs: Polyunsaturated fatty acids
P4HB: Prolyl 4-Hydroxylase Subunit Beta
PDI: Protein Disulfide Isomerase
PE: Phosphatidylethanolamine
PBS: Phosphate Buffered Saline
ROS: Reactive oxygen species
TCGA: The Cancer Genome Atlas
TEM: Transmission electron microscope
TNBC: Triple-negative breast cancer
SLC7A11: Solute Carrier Family 7 Member 11
