# Supplementary material for: CtBP promoted P4HB mediates SLC7A11 maturation to confer ferroptosis resistance in triple-negative breast cancer
Source: J Exp Clin Cancer Res. 2026 May 16;45:153. doi: 10.1186/s13046-026-03727-1 (PMC13348747; doi:10.1186/s13046-026-03727-1)
Supplement: Supplementary file 1 — Supplementary Material 1. [file 13046_2026_3727_MOESM1_ESM.docx]

**Supplementary Files**

**Table S1**

**List of construct primers**

| F-pLKO.1-ERO1A-sg1 | CCGGCTACAAGTGTGAAAGAATTCTCGAGAATTCTTTCACACTTGTAGTTTTTG |
| --- | --- |
| R-pLKO.1-ERO1A-sg1 | AATTCAAAAACTACAAGTGTGAAAGAATTCTCGAGAATTCTTTCACACTTGTAG |
| F-pLKO.1-ERO1A-sg2 | CCGGGTGACTACTTTAGGTATTACTCGAGTAATACCTAAAGTAGTCACTTTTTG |
| R-pLKO.1-ERO1A-sg2 | AATTCAAAAAGTGACTACTTTAGGTATTACTCGAGTAATACCTAAAGTAGTCAC |
| F-lentiv2-P4HB-sg1 | CACCAGGACTCGACTGCCAACGAGG |
| R-lentiv2-P4HB-sg1 | AAACCCTCGTTGGCAGTCGAGTCCC |
| F-lentiv2-P4HB-sg2 | CACCACAAGTACAAGCCCGAATCGG |
| R-lentiv2-P4HB-sg2 | AAACCCGATTCGGGCTTGTACTTGC |
| F-PCDH-P4HB | ATCTCGAGCTCAAGCTTCGGAATTCATGCTGCGCCGCGCTCTGCTGTG |
| R-PCDH-P4HB | TGTAATCCAGAGGTTGATTGGATCCTCAATGATGATGATGATGATGG |
| F-PCDH-P4HB-MUTsite1 | GCCCCTTGGTCTGGCCACTCCAAGGCTCTGGCCCCTGAGTATGCCAAAGC |
| R-PCDH-P4HB-MUTsite1 | CCAGAGCCTTGGAGTGGCCAGACCAAGGGGCATAGAACTCCACCAG |
| F-PCDH-P4HB-MUTsite2 | TATGCCCCATGGTCTGGTCACTCCAAACAGTTGGCTCCCATTTGGGA |
| R-PCDH-P4HB-MUTsite2 | GTCTTTGTGGAGTTCTATGCCCCATGGTCTGGTCACTCCAAACAGTTG |
| R-PCDH-P4HB-ΔKDEL | TGAGTTTTTGTTCGGGCCCAAGCTTCACAGCTTTCTGATCATCGTCT |
| F-pLKO.1-CtBP1/2-sg | CCGGGGGAGGACCTGGAGAAGTTCTCGAGAACTTCTCCAGGTCCTCCCTTTTTG |
| R-pLKO.1-CtBP1/2-sg | AATTCAAAAAGGGAGGACCTGGAGAAGTTCTCGAGAACTTCTCCAGGTCCTCCC |

**List of qPCR primers**

| F-CtBP2 | TTCAAGGCCCTGAGAGTGAT |
| --- | --- |
| R-CtBP2 | GAGTCCGCTGTCTCTTCCAC |
| F-CtBP1 | ACGCTGTGCCACATCCTGAACC |
| R-CtBP1 | AAGAGCACGTTGAAGCCGAAGG |
| F-βACTIN | CACCATTGGCAATGAGCGGTTC |
| R-βACTIN | AGGTCTTTGCGGATGTCCACGT |
| F-SOD1 | TTGCATCATTGGCCGCACACT |
| R-SOD1 | ATTACACCACAAGCCAAACGACT |
| F-SOD2 | AATAGCTGGGATTACAGGTGCAT |
| R-SOD2 | CCAGACGGATCACTTGAGGTCAG |
| F-CAT | CTCCAAATTACTACCCCAACAGC |
| R-CAT | ATCATTGGCAGTGTTGAATCTCC |
| F-NRF2 | CACATCCAGTCAGAAACCAGTGG |
| R-NRF2 | GGAATGTCTGCGCCAAAAGCTG |
| F-GPX1 | CTTCTTGGACAATTGCGCCATG |
| R-GPX1 | CCGAGAAGGCATACACCGAC |
| F-GPX2 | CTACCTGAAGGACAAGCTCCC |
| R-GPX2 | CTTCTCAAAGTTCCAGGCCACA |
| F-GPX4 | ACAAGAACGGCTGCGTGGTGAA |
| R-GPX4 | GCCACACACTTGTGGAGCTAGA |
| F-SLC7A11 | ATGCAGTGGCAGTGACCTTT |
| R-SLC7A11 | GGCAACAAAGATCGGAACTG |
| F-SLC3A2 | CCAGAAGGATGATGTCGCTCAG |
| R-SLC3A2 | GAGTAAGGTCCAGAATGACACGG |
| F-GCLC | GGAAGTGGATGTGGACACCAGA |
| R-GCLC | GCTTGTAGTCAGGATGGTTTGCG |
| F-GCLM | TCTTGCCTCCTGCTGTGTGATG |
| R-GCLM | TTGGAAACTTGCTTCAGAAAGCAG |
| F-PRDX1 | TTCACTGACAAACATGGGGAA |
| R-PRDX1 | CGCTCACTTCTGCTTGGAGA |
| F-ATF3 | CGCTGGAATCAGTCACTGTCAG |
| R-ATF3 | CTTGTTTCGGCACTTTGCAGCTG |
| F-MAP3K14 | GGAATACCTCCACTCACGAAGG |
| R-MAP3K14 | CTGTGAGCAAGGACTTTCCCAG |
| F-PRKAA2 | CCAGAGAATGTCCTGTTGGATGC |
| R-PRKAA2 | CTGAGATGACTTCAGGTGCTGC |
| F-SLC39A14 | CTGGACCACATGATTCCTCAGC |
| R-SLC39A14 | AGAGTAGCGGACACCTTTCAGC |
| F-AKT1S1 | CGGTCATCAGATGAGGAGAATGG |
| R-AKT1S1 | GCTTCTGGAAGTCGCTGGTGTT |
| F-AKR1C3 | CCGAAGCAAGATTGCAGATGGC |
| R-AKR1C3 | GTGAGTTTTCCAAGGCTGGTCG |
| F-NT5DC2 | AGCCTGGAGTTTGACCAAGCAC |
| R--NT5DC2 | CAGGACAGCAAACGTCTCATCC |
| F-NQO1 | CCTGCCATTCTGAAAGGCTGGT |
| R-NQO1 | GTGGTGATGGAAAGCACTGCCT |
| F-PANX2 | CCTCACCATCCTGAGCCGAAAC |
| R-PANX2 | GACGGTGCCATCCCTCAAAACT |
| 1. CDC25A | TCTGGACAGCTCCTCTCGTCAT |
| R-CDC25A | ACTTCCAGGTGGAGACTCCTCT |
| F-SREBF1 | ACTTCTGGAGGCATCGCAAGCA |
| R-SREBF1 | AGGTTCCAGAGGAGGCTACAAG |
| F-FZD7 | GTCTTCAGCGTGCTCTACACAG |
| R-FZD7 | ACGGCATAGCTCTTGCACGTCT |
| F-P4HB | TCACCAAGGAGAACCTGCTGGA |
| R-P4HB | GGCAAGAACAGCAGGATGTGAG |
| F-PDIA2 | GCTGAGGAGTTTGGTGTGAC |
| R-PDIA2 | AGAAGCCAATGACCACTAGGT |
| F-PDIA3 | GTCAGCCACTTGAAGAAGCAGG |
| R-PDIA3 | TAGGAACTCGGAGTGAGCCTCA |
| F-PDIA4 | CCAGCAGGTTTGATGTGAGTGG |
| R-PDIA4 | GGAGACTTCTCTGACCTTGGCA |
| F-PDIA5 | GGAGCCAAAGATGTTGTCCACC |
| 1. PDIA5 | GAAATGCGGCATCATCCTCTTGC |
| F-PDIA6 | TCAGAAAGGCGAGTCTCCTGTG |
| R-PDIA6 | CCTCTTGGCAATGTCCTCGTTG |
| F-TXNDC5 | GTAGACTGCACTGCTGAACGGA |
| R-TXNDC5 | TCGTCTTTCGCTTGGCTCAGGA |
| F-TXNDC12 | GGACATAATGGGCTTGGAAAGGG |
| R-TXNDC12 | CTTTGCAAGCTCCACACCAGGA |
| F-DHFR | CATGGTCTGGATAGTTGGTGGC |
| R-DHFR | GTGTCACTTTCAAAGTCTTGCATG |
| F-GCH1 | GCCATGCAGTTCTTCACCAAGG |
| R-GCH1 | ATGGAACCAAGTGATGCTCACAC |
| F-DHODH | GAGGACATTGCCAGTGTGGTCA |
| R-DHODH | TTCCCACTCAGCCCTCCTGTTT |


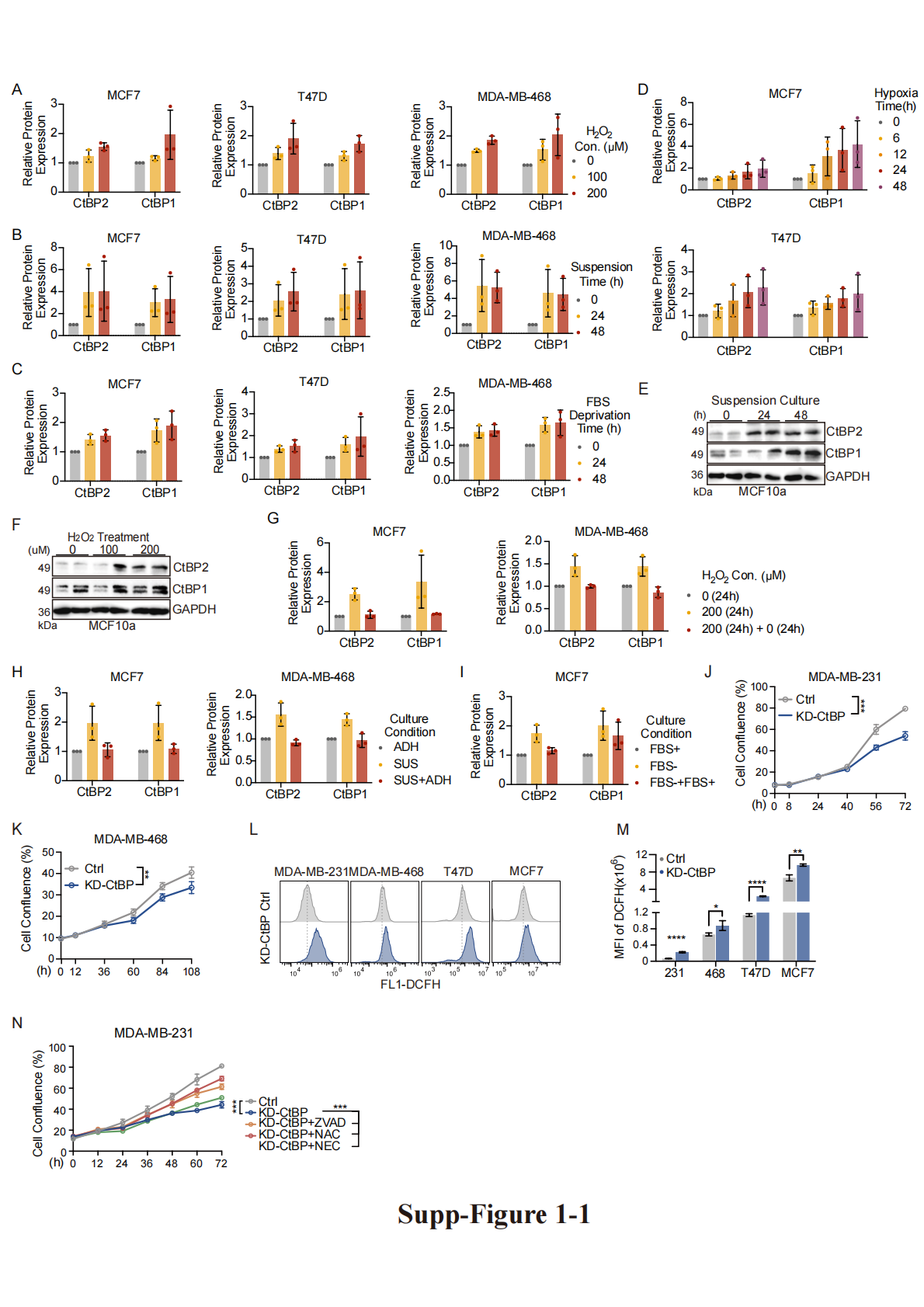
 **Sup Fig 1-1.** (A)Quantification of WB analysis of CtBP in breast cancer cells following treatment with the indicated concentrations of H_2_O_2_ for 24 hours (n=3). (B–D) Quantification of WB analysis of CtBP under suspension culture (B), FBS deprivation (C), and hypoxic culture conditions (D) (n=3). (E) WB quantification of CtBP in MCF10a cells under suspension culture with increasing time. (F) WB quantification of CtBP in MCF10a cells treated with 0, 100, and 200 µM H₂O₂ for 24 hours. (G–I) Quantification of WB analysis of CtBP in breast cancer cells treated with 200 μM H_2_O_2_ (G), suspension culture (H), or FBS deprivation (I) for 24 hours, followed by withdrawal of the treatment and subsequent culturing for another 24 hours (n=3).(J, K) Cell confluence percentage of MDA-MB-231 and MDA-MB-468 cells cultured under adhesion conditions in real time assessed by IncuCyte ZOOM (n=3). (L) Flow cytometry assay of DCFH-DA staining of MDA-MB-231, MDA-MB-468, T47D, and MCF7 cells with CtBP KD (n=3). (M) Histogram showing quantification of Mean Fluorescence Intensity (MFI) of DCFH-DA in the cells described in (L) (n=3). (N) Cell confluence percentage treated with ZVAD (10 µM), NAC (2 mM), or NEC (0.5 µM) for 24 hours (n=3). P values were calculated using a two-tailed Student’s t-test. #, P > 0.05; *, P < 0.05; **, P < 0.01; ***, P < 0.001; ****, P < 0.0001. All data are presented as mean ± SD and (n≥3).

**
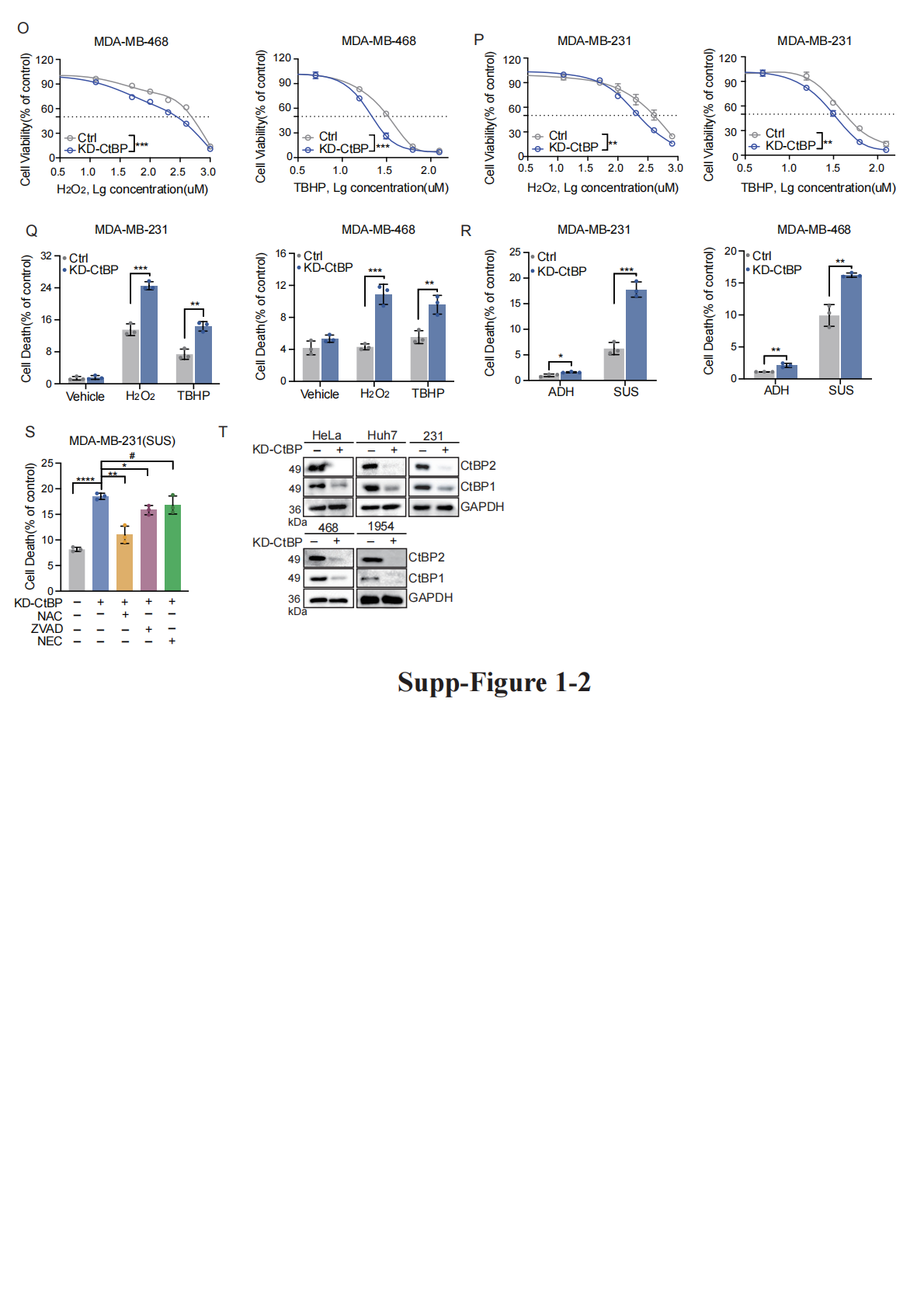
**

**Sup Fig 1-2.** (O, P) Viability of MDA-MB-468 (O) and MDA-MB-231 (P) cells determined by CCK-8 assay after treatment with various concentrations of H₂O₂ or TBHP for 24 hours (n=3). (Q) Cell death percentage (assessed by PI staining) of MDA-MB-231 and MDA-MB-468 cells treated with H₂O₂ (500 µM) or TBHP (30 µM) for 24 hours (n=3). (R) Cell death percentage (assessed by PI staining) of MDA-MB-231 and MDA-MB-468 cells cultured under adhesion or suspension conditions for 24 hours (n=3). (S) Cell death percentage (assessed by PI staining) of MDA-MB-231 cells cultured under suspension conditions and treated with ZVAD (10 µM), NAC (2 mM), or NEC (0.5 µM) for 24 hours (n=3). (T) WB quantification of CtBP knockdown efficiency in HeLa, Huh7, MDA-MB-231, MDA-MB-468, and HCC1954 cells. P values were calculated using a two-tailed Student’s t-test. #, P > 0.05; *, P < 0.05; **, P < 0.01; ***, P < 0.001; ****, P < 0.0001. All data are presented as mean ± SD and (n≥3).

 **Sup Fig 2.** (A) RT-qPCR analysis of CtBP mRNA in MCF7 cells exposed to different stresses including H₂O₂ (24h), Suspension (24h), FBS deprivation (24h) and hypoxia (24h) etc (n=3). (B) and (C) Dosage and time dependent change of CtBP2 mRNA in MCF-7 cells upon hypoxia treatment (n=3). (D) WB quantification of CtBP expression in cells treated with 200 µM H₂O₂ for 24 hours, with treatment by MG132 (20 µM) for 8 hours (n=3). (E) Quantification of CtBP2 mRNA upon hypoxia followed by normoxia recovery as indicated (n=3). (F) WB quantification of CtBP expression in cells treated with 200 µM H₂O₂ for 24 hours, with co-treatment by CHX (30 µg/mL) for the indicated times (n=3). (G) Histogram showing altered abundance of glutathione and its derivatives from metabolomic analysis in MDA-MB-231 cells (n = 6). (H, I) RT-qPCR detection of the expression of key genes involved in the glutathione-independent ferroptosis pathway in MDA-MB-468 and HCC1954 cells (n=3). (J) WB quantification of indicated proteins in MDA-MB-231, MDA-MB-468 and HCC1954 cells with CtBP KD (n=3). (K) Histograms showing relative abundance of major phospholipid classes (PE, PC, and lysophospholipids) and free fatty acids (FA) in CtBP KD versus control MDA-MB-231 cells, determined by metabolomic analysis (n = 6); lipids were classified into polyunsaturated (PUFA) and monounsaturated (MUFA) fatty acids. (L) GSEA enrichment of the hallmark Reactive Oxygen Species (ROS) pathway in breast cancer cells with CtBP KD with RSL3 treatment. (M) Heatmap shows the DEGs from RNA-seq involved in the KEGG ferroptosis pathway (hsa04216) (n = 2). (N) Lollipop charts for GO analysis of RNA-Seq data; DEGs are defined as genes with |Log₂(fold change) | > 0.5 and adjusted p-value < 0.05. (O) RT-qPCR detection of antioxidant gene expression in MDA-MB-231, MDA-MB-468, and HCC1954 cells with CtBP KD (n=3). P values were calculated using a two-tailed Student’s t-test. #, P > 0.05; *, P < 0.05; **, P < 0.01; ***, P < 0.001; ****, P < 0.0001. All data are presented as mean ± SD and (n≥3).

 **Sup Fig 3.** (A) Heatmap shows cell viability (upper) and lipid peroxidation (detected by C11 Bodipy, lower) in MDA-MB-231, MDA-MB-468, BT549, T47D, and MCF7 cells treated with RSL3 (200 nM) and Erastin (25 µM) for 24 hours (n=3). (B) Histogram shows quantification of ROS by Flow cytometry using the DCFH-DA probe in MDA-MB-468 and HCC1954 cells treated with RSL3 (20 nM and 40 nM) and Erastin (25 µM) for 24 hours, with CtBP KD (n=3). (C) Immunofluorescence (IF) staining using CtBP1 and CtBP2 antibodies to detect CtBP expression (green) in MDA-MB-468 parental and RSL3-resistant (RR) cells, as well as MDA-MB-231 parental and Erastin-resistant (ER) cells. (D) Flow cytometry using the DCFH-DA probe to detect cellular ROS in MDA-MB-468 and MDA-MB-231 parental and resistant cells (n=3). (E) Cell viability assessment of MDA-MB-468 and MDA-MB-231 cells treated with increasing concentrations of Erastin and RSL3 (n=3). (F) Flow cytometry using the C11 Bodipy probe to detect lipid peroxidation in MDA-MB-468 and MDA-MB-231 parental and resistant cells treated with RSL3 (200 nM) or Erastin (100 µM) (n=3). (G) WB quantification of CtBP KD efficiency in MDA-MB-468 and MDA-MB-231 parental and resistant cells (n=3). .(H) WB quantification of CtBP2 overexpression (OE) efficiency in MDA-MB-468 cells. P values were calculated using a two-tailed Student’s t-test. #, P > 0.05; *, P < 0.05; **, P < 0.01; ***, P < 0.001; ****, P < 0.0001. All data are presented as mean ± SD and (n≥3).


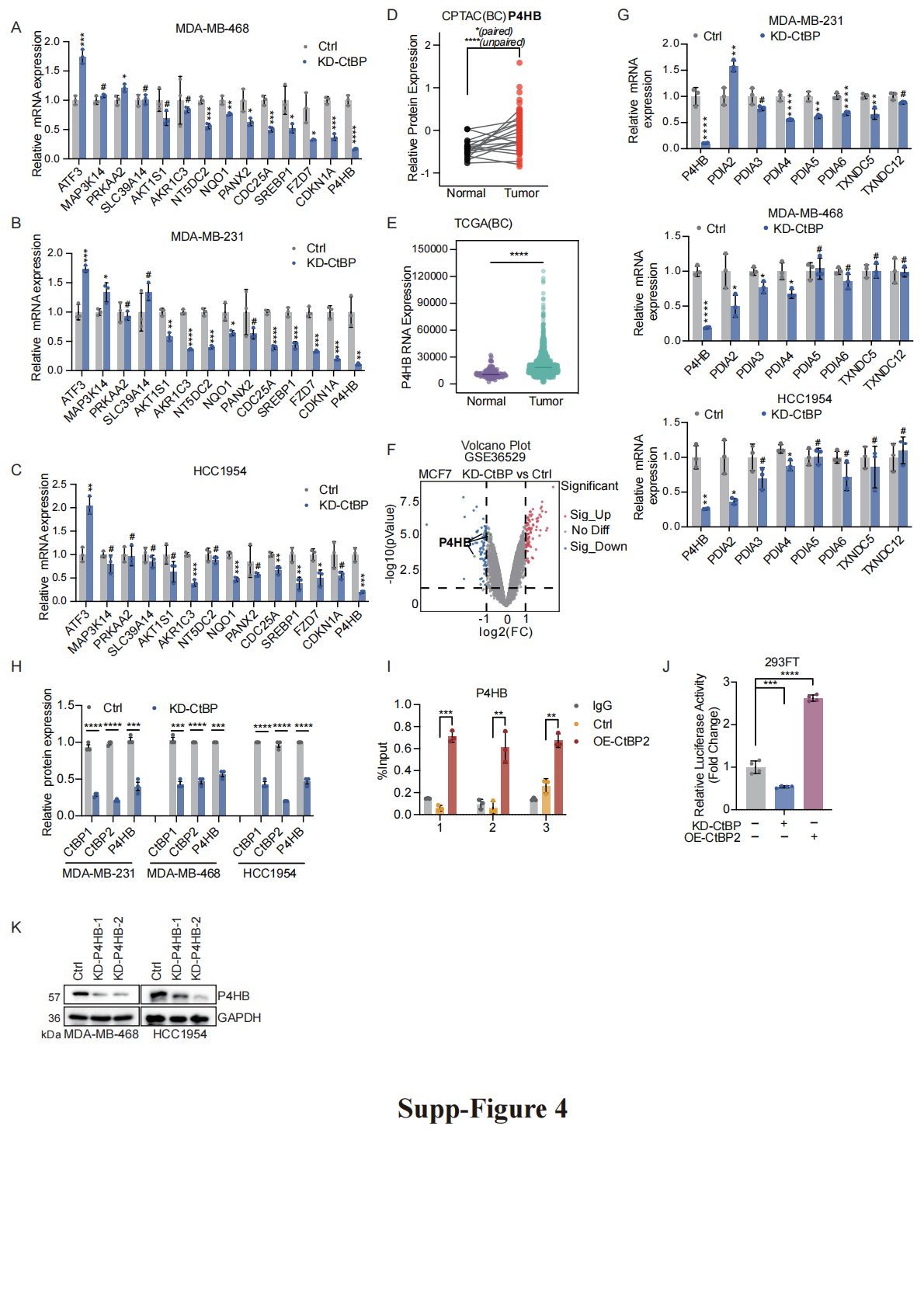
 **Sup Fig 4.** (A-C) RT-qPCR validation of 14 candidate genes expression in MDA-MB-468, MDA-MB-231, and HCC1954 cells with CtBP KD (n=3). (D) Comparison of P4HB protein level expression in breast cancer samples (n=124) and matched normal mammary gland tissues (n=18), data retrieved from the CPTAC dataset. (E) Comparison of P4HB mRNA levels in breast cancer (n=1097) and adjacent normal tissues (n=113), data retrieved from TCGA. (F) Volcano plot of DEGs in MCF7 cells with CtBP KD, data retrieved from the GSE36529 RNA-Seq dataset; green dots indicate P4HB. DEGs are defined as genes with |Log₂ (fold change) | > 1 and adjusted p-value < 0.05. (G) RT-qPCR detection of the expression of PDI family members in MDA-MB-231, MDA-MB-468, and HCC1954 cells with CtBP KD (n=3). (H) Quantification of WB analysis of P4HB in breast cancer cells with CtBP KD (n=3). (I) ChIP-seq analysis of CTBP2 in 293FT cells with three primer pairs targeting CTBP2 binding peak at the P4HB promoter (n=3). (J) Dual-luciferase reporter assays in 293FT cells with CtBP knockdown or CtBP2 overexpression (n=3). (K) WB quantification of P4HB KD efficiency in MDA-MB-468 and HCC1954 cells. P values were calculated using a two-tailed Student’s t-test. #, P > 0.05; *, P < 0.05; **, P < 0.01; ***, P < 0.001; ****, P < 0.0001. All data are presented as mean ± SD and (n≥3).

 **Sup Fig 5.** (A) WB quantification of the expression of indicated proteins in MDA-MB-468 and HCC1954 cells with CtBP KD in combination with P4HB-Myc OE (n=3). (B) Histogram shows the ROS quantification by Flow cytometry using DCFH-DA probe in MDA-MB-468 and HCC1954 cells (n=3). (C) WB quantification of the expression of P4HB, and P4HB mutants in MDA-MB-468 and HCC1954 cells (n=3). (D) Fluorescence images showing the cellular localization of full-length P4HB, Mut-P4HB, and ΔKDEL-P4HB (red); the ER was stained with ER-tracker (blue) in MDA-MB-468 cells. (E, F) WB analysis using the indicated antibodies in MDA-MB-468 cells (n=3). (G) H&E staining of lung sections, with metastatic tumor colonies indicated by red circles. P values were calculated using a two-tailed Student’s t-test. #, P > 0.05; *, P < 0.05; **, P < 0.01; ***, P < 0.001; ****, P < 0.0001. All data are presented as mean ± SD and (n≥3).
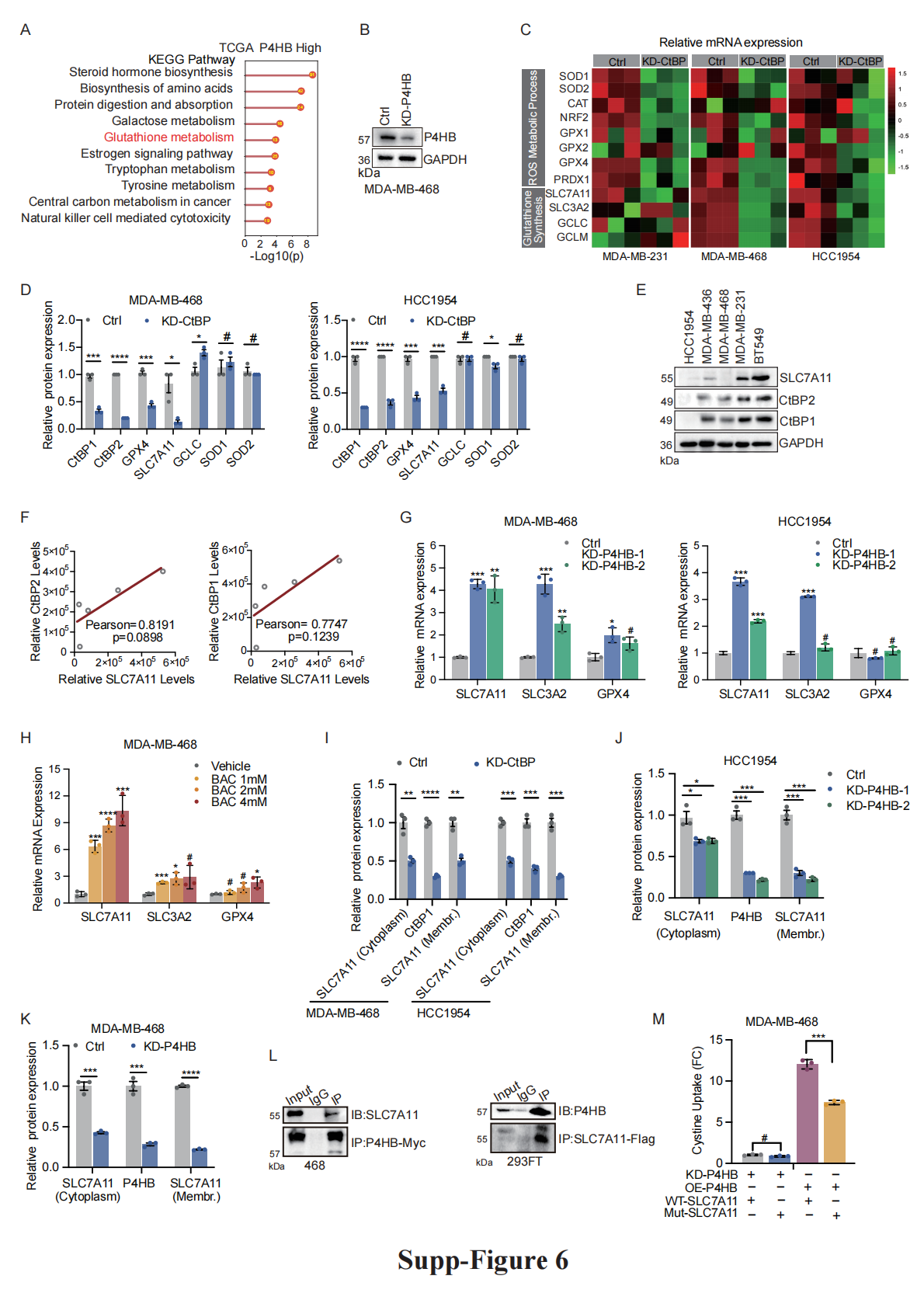
 **Sup Fig 6.** (A) KEGG Pathway analysis of DEGs from P4HB-high samples in TCGA breast cancer data; patients were categorized into high- and low-P4HB expression groups using the median expression level as the threshold. DEGs are defined as genes with |Log₂(fold change)| > 0.5 and adjusted p-value < 0.05. (B) WB quantification of P4HB knockdown efficiency in MDA-MB-468 cells. (C) Heatmap showing the expression of genes involved in the ROS metabolic process and glutathione synthesis measured by RT-PCR in MDA-MB-231, MDA-MB-468, and HCC1954 cells with CtBP KD (n=3). (D) Quantification of WB analysis of indicated proteins in breast cancer cells with CtBP KD (n=3). (E) WB analysis using the indicated antibodies in five TNBC cell lines. (F) Dot plot showing the correlation of SLC7A11 with CtBP1 or CtBP2 (determined by Western blotting) in five TNBC cell lines. (G) RT-qPCR detection of the expression of SLC7A11, SLC3A2, and GPX4 in MDA-MB-468 and HCC1954 cells with P4HB KD (n=3). (H) RT-qPCR detection of the expression of SLC7A11, SLC3A2, and GPX4 in MDA-MB-468 cells treated with increasing concentrations of bacitracin for 24 hours (n=3). (I-K) Quantification of WB analysis of membrane and cytosolic fractions of indicated proteins (n=3). (L) Reciprocal co-immunoprecipitation assays in MDA-MB-468 or 293FT cells overexpressing P4HB-Myc or SLC7A11-Flag. (M) Cystine uptake assay in P4HB-deficient or wild-type MDA-MB-468 cells overexpressing wild-type SLC7A11 or its C158-deficient mutant (n=3). For (F), P values were calculated using a Pearson correlation test. Other P values were calculated using a two-tailed Student’s t-test. #, P > 0.05; *, P < 0.05; **, P < 0.01; ***, P < 0.001; ****, P < 0.0001. All data are presented as mean ± SD and (n≥3).

 **Sup Fig 7.** (A-C) Correlation between ferroptosis-related suppressor score or driver score and the relative abundances of CtBP1 and P4HB in breast cancer (A, C) and TNBC (B); ferroptosis suppressor and driver score were referenced from FerrDb, and scores were analyzed using ssGSEA followed by a Pearson correlation test. (D) Semi-quantification of IHC scores of CtBP2 and SLC7A11 in TNBC sections (n = 48), analyzed using a Pearson correlation test.
